# Re-examination of the Claimed Isolation of Stable Noncyclic 1,2-Disulfoxides

**DOI:** 10.1021/acs.orglett.4c02849

**Published:** 2024-09-04

**Authors:** Eric Block, Julien J. H. Cotelesage, Evgeny Dikarev, Benedetta Garosi, Graham N. George, Rabi A. Musah, Linda I. Vogt, Zheng Wei, Yuxuan Zhang

**Affiliations:** # Department of Chemistry, University at Albany, State University of New York, Albany, New York 12222, United States; ‡ Molecular and Environmental Sciences Group, Department of Geological Sciences, University of Saskatchewan, Saskatoon, Saskatchewan S7N 5E2, Canada

## Abstract

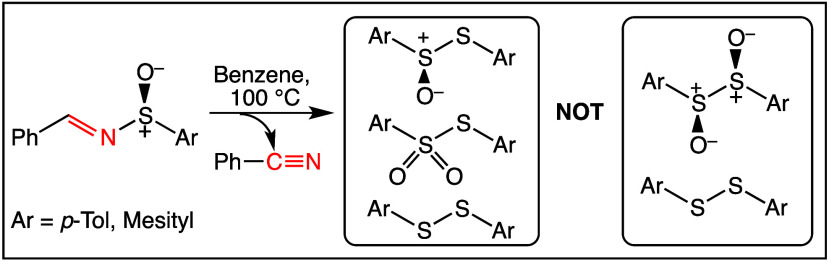

Re-examination of the claimed isolation and X-ray characterization of di-*p*-tolyl and dimesityl 1,2-disulfoxides from thermolysis of the corresponding aryl sulfinimines and thiosulfinates showed that the isolated disulfide dioxides are instead the well-known isomeric thiosulfonates, as confirmed by XAS, DART-MS, X-ray, IR and NMR methods. Concerns with the original X-ray structures are addressed. Our results agree with the DFT prediction of very weak diaryl 1,2-disulfoxide S–S bond dissociation enthalpies. For now, room-temperature-stable noncyclic 1,2-disulfoxides remain unknown.

For more than 150 years the structural distinction of 1,2-disulfoxides (α- or *vic*-disulfoxides) and isomeric thiosulfonates from oxidation of disulfides has been the subject of recurrent investigation (see timeline in the Supporting Information (SI)). Most publications identify the structure of acyclic disulfide dioxides as thiosulfonates rather than 1,2-disulfoxides (e.g., sulfonyl IR and Raman bands favor the thiosulfonate formulation),^1−3^ simultaneously affirming the considerable instability of acyclic 1,2-disulfoxides. Kice, in his low temperature ^19^F NMR study of the oxidation of *p*-fluorophenyl thiosulfinates, suggests a half-life for diaryl 1,2-disulfoxides of less than 60 s at −20 °C, indicating that such compounds are “extraordinarily unstable”.^4^ The *bis*-1-alkenyl 1,2-disulfoxide onion extract intermediate is equally unstable, immediately undergoing [3,3]-rearrangement at −20 °C to a *bis*-sulfine.^5,6^ Jenks, in his computational study of MeS(O)S(O)Me, reports a S–S bond dissociation enthalpy (BDE) of 20 kcal mol^–1^, asserting that “the weakness of the S(O)–S(O) bond is derived from the unusual stability of the two product sulfinyl radicals”,^7^ as Kice earlier suggested.^4^

A cyclic 1,2-disulfoxide with the highest stability so far reported, *trans*-2,7-di-*tert*-butylnaphtho-[1,8-*cd*]dithiole 1,2-dioxide (*trans*-**1**; Figure 1), places the disulfoxide in a five-membered ring bridging the *peri*-positions of naphthalene. Formation of the disulfide bridge is favorable, reducing *peri*-interactions. Introduction of the bulky *tert*-butyl groups forces the two sulfur atoms closer together, disfavoring S–S homolysis. When S–S homolysis does occur during racemization of enantiomers of *trans*-**1**, it is followed by S–S recombination, favored over head-to-tail S–O coupling of disulfinyl diradical **2** due to unfavorable steric effects associated with the *peri*-fused six-membered *O*,*S*-sulfenyl sulfinate ring **3**.^8,9^

**Figure 1:**
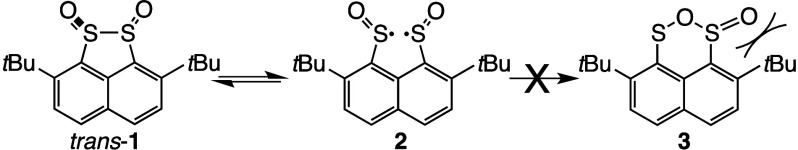
Stable cyclic 1,2-disulfoxide *trans*-**1**.

Unsurprisingly, an X-ray structure of *trans*-**1** shows a significantly shorter S–S bond than that in other known, less stable cyclic 1,2-disulfoxides.^9^

In considering the enhanced stability of cyclic compared to acyclic 1,2-disulfoxides, it is informative to compare S–S bond rupture in cyclooctasulfur, S_8_, and tetrasulfane, HSS–SSH. Thus, while S_8_ has a slightly lower S–S bond enthalpy (S_8_ Δ*H*_0_ = 40.5 kcal/mol, H_2_S_4_ Δ*H*_0_ = 41.8 kcal/mol), this is insufficient to make S_8_ the better thermal source of XS^•^ radicals due to entropic factors (S_8_ Δ*G*_0_ = 35.5 kcal/mol and H_2_S_4_ Δ*G*_0_ = 29.6 kcal/mol). The TΔ*S* term for ring opening of S_8_ is 5.5 kcal/mol compared to 13.6 kcal/mol for dissociation of HSSSSH to 2HSS^•^. The latter process affords two independent species while S_8_ gives a tethered diradical similar to **2**.^10^

Diaryl thiosulfonates, useful reagents for organic synthesis,^11,12^ are products of disproportionation of diaryl thiosulfinates, of reaction of arenesulfinyl chlorides with Zn or Cu,^13,14^ and of thermolysis of alkylidene arenesulfinimines^15^ (RCH = NS(O)Ar). As already noted, while *cyclic* 1,2-disulfoxides are isolable, moderately stable species,^9^ noncyclic 1,2-disulfoxides have thus far eluded room temperature isolation and have only been detected by low temperature NMR methods.^16−19^

With the above as background, the 2014 publication by Stockman’s group^20^ was therefore notable in its claim to have “unambiguously confirmed” by X-ray analysis the structures of two diaryl noncyclic 1,2-disulfoxides whose “instability was overestimated.” In particular, it was asserted that 1974 work by Davis and co-workers (Figure 2)^15^ showing that thermolysis of sulfinimines (**4**) for 20 h at 100 °C afforded diaryl thiosulfonates (**7**) and disulfides (**8**) via thermal disproportionation of diaryl thiosulfinates (**6**) was incorrect, and instead affords isolable 1,2-disulfoxides (**9**). Davis’s observation of the disproportionation of **6** was consistent with earlier studies by Barnard, Fava, and Oae,^21−24^ as well as studies on alkyl thiosulfinates by one of us.^25,26^ The Stockman group’s 2014 publication has been cited multiple times, yet none of the citing papers question their remarkable conclusions. Surprisingly, the 2014 publication did not compare the chromatographic and spectroscopic properties claimed for their 1,2-disulfoxides with those of the corresponding well-known thiosulfonates. To us, the cited spectroscopic properties seemed suspiciously similar to those reported for thiosulfonates **7a,b** (see SI), while at the same time the X-ray data, which was the sole basis for claiming formation of **9a,b**, seemed problematic.

**Figure 2:**
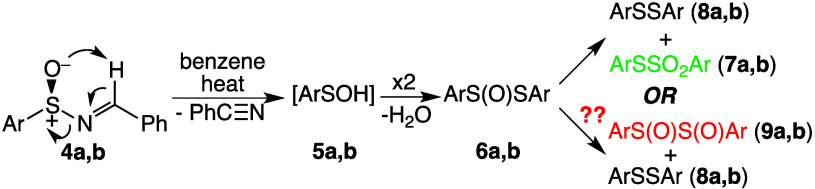
Thermolysis of *N*-benzylidenearenesulfinimines **4**. For Ar in **4**–**9**, **a** = *p*-tolyl and **b** = mesityl.^15,20^

Since earlier computational studies have only modeled acyclic dialkyl disulfoxides,^7^ we performed density functional theory (DFT) calculations using a hybrid PBE0 functional for dialkyl, diphenyl and di-*p*-tolyl 1,2-disulfoxides (**9a**) and related compounds. Encouraged by the agreement of the computations on dimethyl 1,2-disulfoxide with Jenks’s earlier computations and by the even weaker S–S bonding we found for the diaryl 1,2-disulfoxides (Table 1), we repeated the experimental work described in the Stockman group’s 2014 paper including obtaining crystals for studies by X-ray crystallographic and XAS. We report herein the findings together with our interpretation of the mechanism for the reactions initially described in 1974 by Davis as reinvestigated by Stockman’s group.^20^

**Table 1:** Comparative Computed Bond Dissociation Enthalpies (BDEs; kcal/mol) for Dialkyl and Diaryl Disulfides and their *S*-Oxides

compound	this work S–S BDE	Gregory,^7^ Gadde^34^ S–S BDE
MeS(O)S(O)Me *syn*	18.83	∼20^7^
MeS(O)S(O)Me *anti*	16.69	
MeS(O)SMe	39.31 (46^a^)	47^7^
MeSO_2_SMe	54.56	
MeSSMe	63.18 (75^a^)	74^7^
*n*-BuSSBu-*n*		61.93^c^
PhSO_2_SBu-*n*		50.31^c^
PhS(O)S(O)Ph *syn*	13.56	
PhS(O)S(O)Ph *anti*	11.52	
PhS(O)SO_2_Ph	27.61	(27.6)^b^
PhS(O)SPh	27.98	(34.5)^b^
PhSO_2_SO_2_Ph	38.20	(40.9)^b^
PhSO_2_SPh	47.77	
PhSSPh	47.76	
*p*-TolS(O)S(O)Tol-*p*, *syn*-**9a**	13.48	
*p*-TolS(O)S(O)Tol-*p*, *anti*-**9a**	11.80	
*p*-TolS(O)SO_2_Tol-*p*	26.54	
*p*-TolS(O)STol-*p,***6a**	31.34	
*p*-TolSO_2_SO_2_Tol-*p*		39.91^c^
*p*-TolSO_2_STol-*p,***7a**	46.72	43.28^c^
*p*-TolSSTol-*p,***8a**	45.56	45.39^c^

a From mass spectrometric appearance potential measurements.^25^

b Parenthetical values cited by Gregory are from Kice^4^ and Fava.^23^

c Gadde;^34^ Gaussian16.

Table 1 shows calculated S–S BDEs for dialkyl and diaryl disulfides and their *S*-oxides, with a comparison to Jenks’s estimates. Our calculations of BDEs of 19 and 17 kcal/mol for *syn*- and *anti*-MeS(O)S(O)Me, respectively, are in good agreement with the Jenks’s estimated BDE of 20 kcal/mol. Given the significantly lower BDE calculated for PhSSPh (48 kcal/mol) and *p*-TolSSTol-*p* (**8a**; 46 kcal/mol) compared to MeSSMe (63 kcal/mol), it is not surprising that the calculated BDEs for the individual stereoisomers of di-*p*-tolyl 1,2-disulfoxide **9a** are extremely low: 11.8 kcal/mol for the *anti*-isomer and 13.5 kcal/mol for the *syn*-isomer. These values, among the lowest known for covalent bonds, make the isolation of diaryl 1,2-disulfoxides as room-temperature stable compounds following heating for 20 h at 100 °C highly improbable. Diaryl sulfinyl sulfones, calculated to have a S–S BDE of 27.6 kcal/mol, 14 kcal/mol higher than that calculated for *syn*-diaryl 1,2-disulfoxides, can only be stored without decomposition at −18 °C.^27^ The weak S–S bonds in diaryl thiosulfinates (**6**; 31 kcal/mol), sulfinyl sulfones and 1,2-disulfoxides (**9**) are all thought to be the result of the unusual stability of the π-type sulfinyl radicals.^27−29^

We then repeated the thermolysis of (±)-*N*-benzylidene-*p*-toluenesulfinimine (**4a**) as described by Stockman’s group.^20^ Thus, **4a** was heated to reflux in benzene for 15 h, solvent was removed in vacuo, and the residue fractionated by chromatography giving benzonitrile, di-*p*-tolyl disulfide (**8a**), and a crystalline solid, mp 77–78 °C, with a high-resolution mass of 279.0514 (corresponding to [C_14_H_14_O_2_S_2_ + H^+^]) which could either be *p*-tolyl *p*-toluenethiosulfonate (**7a**) or *p*-tolyl 1,2-disulfoxide (**9a**). Compound **9a** would be expected to exhibit chirality at both sulfur atoms, resulting in a mixture of diastereomers (a pair of enantiomers and a *meso* compound, e.g., *R*,*R*/*S*,*S* and *S*,*R*).^8^ To determine whether the product with the formula C_14_H_14_O_2_S_2_ was comprised of a mixture of stereoisomers, the crystals were subjected to chiral TLC analysis. Only a single band was observed, suggesting either that the formula corresponded to the achiral thiosulfonate **7a** or the achiral *meso*-disulfoxide **9a**. An X-ray crystal structure showed it to be thiosulfonate **7a**, whose structural features agreed with those found in several previous X-ray structures of the same compound,^30−33^ as summarized in Table S2.

We also repeated the thermolysis, as described by Stockman’s group^20^ of *p*-tolyl *p*-toluenethiosulfinate (**6a**) in refluxing benzene for 15 h. Solvent was removed in vacuo, and the residue fractionated by chromatography giving di-*p*-tolyl disulfide (**8a**), and a crystalline solid identical to thiosulfonate **7a**. In addition, we refluxed for 15 h a benzene solution of (±)-*N*-benzylidene-2,4,6-trimethylbenzenesulfinimine (**4b**), analogous to the (±)-*N*-cyclohexylmethylidene-2,4,6-trimethylbenzenesulfinimine used by Stockman’s group.^20^ After removing solvent and subjecting the residue to preparative TLC, we isolated mesityl disulfide (**8b**), mesityl thiosulfinate (**6b**), and a crystalline solid, mp 135–136 °C, with a high-resolution mass of 335.1130 (corresponding to [C_18_H_22_O_2_S_2_ + H^+^]) which could either be mesityl 2,4,6-trimethylbenzenethiosulfonate (**7b**) or mesityl 1,2-disulfoxide (**9b**). An X-ray crystal structure revealed it to be **7b**. Structural features are summarized in Table S3. The X-ray structure for this compound has not been previously reported. The compound crystallizes in space group *P*-1 with two crystallographically independent molecules. In one of the molecules two oxygens are positionally disordered over two sulfur atoms with a ratio of 0.89:0.11. There is no disorder detected in the other molecule. The main bond distances and angles are listed in Table S3. Its geometry is quite similar to that of its *p*-tolyl analog **7a**.

The features of the X-ray crystal structures that would be anticipated to be present in **9a** (*p*-TolS(O)-S(O)Tol-*p*) and **9b** (MesS(O)-S(O)Mes) are a consequence of the inversion center at the middle of both molecules (i.e., between the two sulfur atoms). This inversion center defines the asymmetric unit (i.e., the smallest unit that is repeated in the crystal structure) as half of the molecule (either the *p*-TolS(O)- or MesS(O)-moieties), with the other half created by the symmetry operation of the inversion. If the functional group that is present is a sulfoxide unit, then ideally, the asymmetric unit would have a single oxygen atom attached to sulfur, and ideally, in a well-refined crystal structure, this would be indicated by the presence of a positive electron density peak at sulfur which can be refined as an oxygen atom with an occupancy of 1. On the other hand, the observation of two electron density peaks associated with a sulfur atom would indicate the presence of two attached oxygens (i.e., a sulfone moiety) with an occupancy of 1 for each of these two oxygen atoms.

In the asymmetric units of both **9a** and **9b** reported by Stockman’s group,^20^ two strong positive electron density peaks around the sulfur atoms in the asymmetric unit were identified, with occupancies of 0.597 and 0.403 in **9a** and 0.545 and 0.455 in **9b** (adding to 1 and indicating 100% occupancy in each case). The authors identified both as oxygen atoms and interpreted their results as being indicative of the positional disorder of one oxygen atom over two positions around the sulfurs (i.e., either one or the other peak appears, but with neither coexisting at the same time (hence the disorder)).

One way to confirm this interpretation is to compare the sizes of the thermal ellipsoids of the various non-hydrogen atoms in the structure. If the aforementioned occupancies were correctly interpreted to be indicative of the presence of a single oxygen atom attached to each sulfur, then the size of the thermal ellipsoids of the various atoms should be about the same because in a well-refined crystal structure, it is expected that all non-hydrogen atoms should possess comparable isotropic displacement factors (*U*_eq_). However, Stockman group’s^20^ results reveal that *the U*_eq_*values of the two oxygens were approximately twice that of the other non-hydrogen atoms*, indicating a discrepancy for which there are two possibilities: one is that the atoms representing the two electron density peaks on sulfur are misassigned as oxygen but are actually some element much lighter than oxygen (which is untrue since no such elements were involved in the synthesis); the second is that the actual occupancies for the two oxygens are actually much smaller than the values defined in the structure models that were reported. Generally, the observation of such a discrepancy prompts the performance of further investigations to confirm the validity of the refined structure model.^35^ However, this step was not reported by the Stockman group^20^ In our work, when the occupancy factors for the two oxygen atoms were refined independently without any restraints/constraints, a considerable decrease of their values was observed for both structures and the magnitude of *U*_eq_ for the two oxygen atoms became normal. This finding argues against 1,2-disulfoxide structures for **9a** and **9b**. Thus, for **9a** and **9b**, we propose that the reported abnormally large equivalent isotropic displacement factors *U*_eq_ for the two oxygen atoms are likely because their actual occupancy factors are lower than their defined values in the reported structure models,^36^ with the combined oxygen occupancy being lower than unity. In addition, S=O bond distances in the structures reported^20^ for **9a** and **9b** are abnormal. In **9a**, the two S=O bond distances are 1.261(7) Å and 1.379(6) Å and in **9b**, 1.392(6) Å and 1.468(5) Å, with the first three values being abnormally short. A comprehensive search of the Cambridge Structural Database (CSD) was performed, and it was found that the typical S=O distance in most reported structures is between 1.4 and 1.5 Å, further reinforcing doubts about the reported structures.

To further assess the Stockman group’s interpretation of their X-ray data, and to determine whether an alternative treatment would yield results more in alignment with the thiosulfonate structures observed by us and others, we carefully examined the. cif files reported by the Stockman group for both **9a** and **9b**.^20^ In their work, the structure models of both disulfoxides were refined against the raw data embedded in the. cif files. However, instead of restraining the sum of the occupancy factors of the two oxygen atoms to unity as was done by the Stockman group, we refined the occupancy factors for the two oxygen atoms independently without applying any constraints/restraints. This resulted in a considerable decrease in the O:S ratios for both structures. In **9a**, the O:S ratio dropped from 2:2 to 1.3:2 and in **9b**, it dropped to 1.5:2. Furthermore, the *U*_eq_ values for the oxygen atoms more reasonably compared to those of the other non-hydrogen atoms in the molecule. This treatment also resulted in a dramatic drop in the *R*-values (for **9a**, a drop from *R*_1_ = 5.15% and *wR*_2_ = 14.37% to *R*_1_ = 3.99% and *wR*_2_ = 10.83%; for **9b**, a drop from *R*_1_ = 5.68% and *wR*_2_ = 17.48% to *R*_1_ = 5.19% and *wR*_2_ = 16.50%). These findings yielded formulas for **9a** and **9b** of C_14_H_14_O_1.3_S_2_ and C_14_H_14_O_1.5_S_2_ which better fit with the experimental data (and fully support thiosulfonate structures), rather than the formulas of C_14_H_14_O_2_S_2_ for **9a** and C_18_H_22_O_2_S_2_ for **9b** that were proposed by the Stockman group.^20^

We have also confirmed the nonidentity of the two sulfur atoms in **7a** and **7b** using sulfur K-edge X-ray absorption spectroscopy (XAS). Sulfur K-edge XAS is very sensitive to electronic structure, allowing different sulfur functionalities to be distinguished. Spectra of both solids and toluene solutions are very similar (not illustrated). We compared the experimental sulfur K-edge XAS with DFT spectral simulations, shown in Figure 3 for **8a**, **6a**, **7a**, and **9a**. The agreement between the DFT simulated spectra for known compounds was found to be excellent, and an entirely different spectrum is predicted for the disulfoxide (**9a**), clearly indicating that the reaction products are aryl thiosulfonates and not the 1,2-disulfoxides.

**Figure 3:**
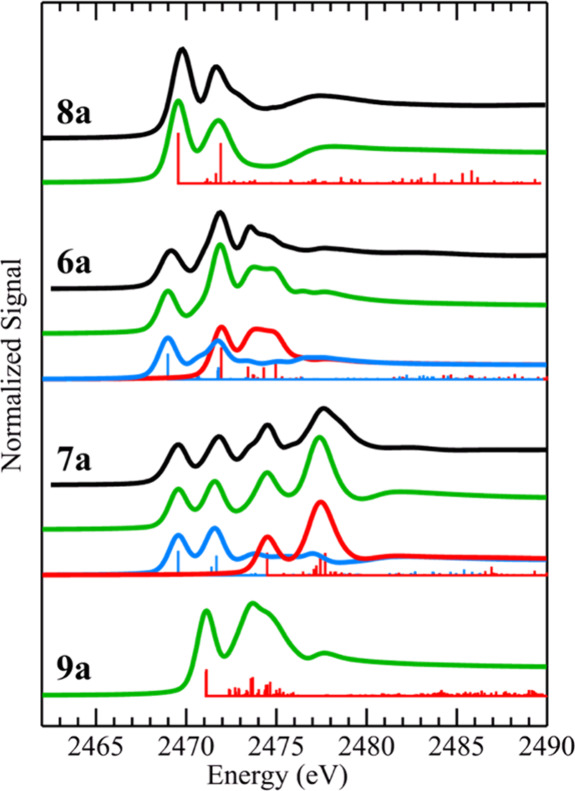
Sulfur K-edge XAS of the *p*-tolyl series **8a** (disulfide), **6a** (thiosulfinate), **7a** (thiosulfonate). The black lines show experimental spectra, while the green lines show the DFT spectral simulations. Compounds **6a** and **7a** contain sulfur in two different formal oxidation states, and the predicted spectra of these different sulfurs are shown by the blue (reduced) and red (oxidized) lines along with stick spectra showing the DFT computed transition energies and intensities. The predicted spectrum of 1,2-disulfoxide **9a** is shown for comparison and can be seen to be distinct from all of the experimental spectra. Similar results were found for the mesityl series **8b**, **6b**, and **7b** (not shown).

The SI includes compilations of infrared absorptions and ^1^H and ^13^C NMR chemical shifts from our work and the chemical literature for dimesityl disulfide (**8b**), thiosulfonates **7a** and **7b**, and thiosulfinate **6b**, along with the published values from the Stockman group^20^ of the claimed 1,2-disulfoxides **9a** and **9b**.^20^ In particular, we note that their IR and NMR values for **9a** and **9b** are closely similar to the values for **7a** and **7b**, respectively. In summary, all of the data suggest that Stockman group’s disulfoxides **9a** and **9b** are in fact thiosulfonates **7a** and **7b**, respectively.

Based on our DFT calculations and earlier literature, we propose the overall mechanism given in Figure 4 for decomposition of sulfinimines **4a**,**b**. After loss of benzonitrile, sulfenic acids **5a**,**b**, sulfinic acids **10a**,**b** and thiosulfinates **6a**,**b** (**6b** is isolable) are detected by DART-HRMS. None of these compounds have been previously found from decomposition of **4a**,**b**. Isolable compounds **7a**,**b** and **8a**,**b** also form, the only other organosulfur compounds seen. Disproportionation of diaryl thiosulfinates **6a,b** likely involves formation and reactions of sulfenyl and sulfinyl radicals as previously proposed.^37^

**Figure 4:**
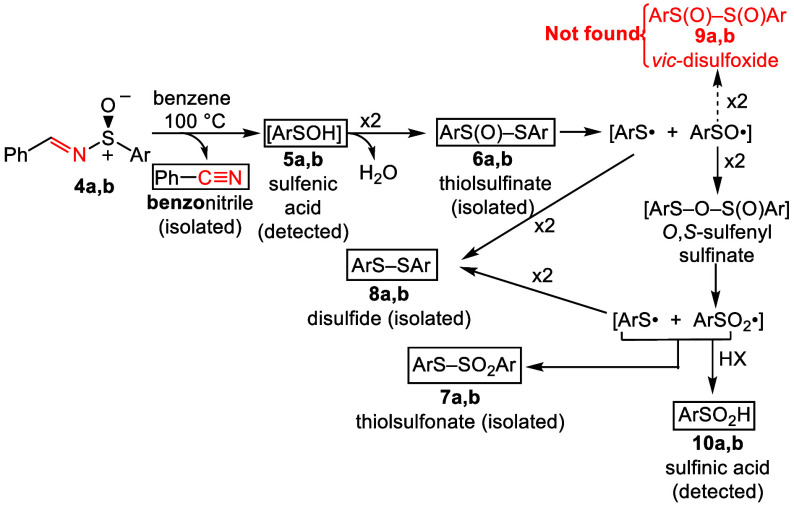
Radical disproportionation pathways for diaryl thiosulfinates (**a** = *p*-tolyl, **b** = mesityl).

In summary, in our opinion the X-ray data reported by Stockman’s group do not support 1,2-disulfoxide structures for the diaryl disulfide dioxides they isolated. We conclude that their conclusions are incorrect based on the following: 1) the similarity of the spectral features of Stockman group’s diaryl disulfide dioxides with those reported for the analogous, known diaryl thiosulfonates; 2) the absence of evidence directly distinguishing the Stockman group’s diaryl disulfide dioxides from the analogous diaryl thiosulfonates; 3) our experimental results repeating the Stockman group’s work; 4) our computational results; and 5) the extensive data from the chemical literature favoring the diaryl thiosulfonate structure over the diaryl 1,2-disulfoxide structure. For the present, room-temperature-stable noncyclic 1,2-disulfoxides remain unknown.

## Data Availability

The data underlying this study are available in the published article and its Supporting Information.

## Supporting Information Available

Experimental procedures, spectroscopic data (^1^H NMR, ^13^C NMR, IR, HRMS, XAS), X-ray structural data, DFT calculations, comparison of ^1^H, ^13^C, and IR data for *p*-tolyl and mesityl disulfides and *S*-oxides (eleven tables), and an historical summary of 1,2-disulfoxides (PDF)
