# Supplementary material for: Re-examination of the Claimed Isolation of Stable Noncyclic 1,2-Disulfoxides
Source: Org Lett. 2024 Sep 4;26(45):9619–24. doi: 10.1021/acs.orglett.4c02849 (PMC11574841; doi:10.1021/acs.orglett.4c02849)
Supplement: Supplementary file 1 — ol4c02849_si_001.pdf [file ol4c02849_si_001.pdf]

Supporting Information

Re-examination of the Claimed Isolation of Stable Non-cyclic 1,2-Disulfoxides

Eric Block,*# Julien J. H. Cotelesage,‡ Evgeny Dikarev,*# Benedetta Garosi,# Graham N. George,*‡ Rabi A. Musah,*# Linda I. Vogt,‡ Zheng Wei,# and Yuxuan Zhang#

#Department of Chemistry, University at Albany, State University of New York, Albany, NY 12222, United States
‡Molecular and Environmental Sciences Group, Department of Geological Sciences, University of Saskatchewan, Saskatoon, Saskatchewan S7N 5E2, Canada

eblock@albany.edu; edikarev@albany.edu; g.george@usask.ca; rmusah@albany.edu

Table of Contents

Materials and methods……………………………………………………………………….2

Synthesis of disulfides……………………………………………………………………….3

Synthesis of thiosulfinates……………………………………………………………..........3

Synthesis of sulfinimines……………………………………………………………...…..5

Thermolysis reactions………………………………………………………………………..6

Synthesis of thiosulfonates from thiosulfinates……………………………………......….8

Infrared spectra………………………………………………………………………………9

¹H-NMR and ¹³C-NMR Spectra……………………………………………………….……13

Crystal structure solution and refinement……………………………………………......20

Figure S1. The molecular structure of di-tolyl thiosulfonate at 100(2) K…………….………...…21

Figure S2. The molecular structure of di-mesityl thiosulfonate at 100(2) K……………………...22

Table S1. Crystallographic data of di-tolyl thiosulfonate and di-mesityl thiosulfonate.……….…23

Table S2. Bond distances and angles (Å, º) for di-tolyl thiosulfonate…………………………….24

Table S3. Bond distances and angles (Å, º) for di-mesityl thiosulfonate………….………………26

X-ray absorption spectroscopy…………………………………………………………...30

Density functional theory (DFT) geometry optimizations………………………………..30

Density functional theory simulations of sulfur near-edge spectra……………………………….31

Table S4. Geometry optimized cartesian coordinates and total energies for the compounds listed in Table 1, plus free radicals required for calculation of BDE values……………………………34
Table S5. Comparison of ^{1}H NMR data (ppm) for di-*p*-tolyl thiosulfinate………………………53
Table S6. Comparison of ^{13}C and ^{1}H NMR data for dimesityl disulfide………………………...53
Table S7. Comparison of ^{13}C NMR data (ppm) for *p*-TolSO$_2$STol-*p* and Tol "disulfoxide"…….53
Table S8. Comparison of ^{1}H NMR data (ppm (*J* Hz)) for *p*-TolSO$_2$STol-*p* and Tol "disulfoxide..54
Table S9. Comparison of ^{13}C NMR data (ppm) for MesSO$_2$SMes and Mes "disulfoxide"……….54
Table S10. Comparison of ^{1}H NMR data (ppm) for MesSO$_2$SMes and Mes "disulfoxide"……………………………………………………………………………..54
Table S11. Comparison of IR data (cm^{-1}) for *p*-TolSO$_2$STol and Tol "disulfoxide"…………....55
Table S5-S11 References……………………………………………………………...56
Historical summary of 1,2-disulfoxides, RS(O)S(O)R, and relationship to thiosulfonates, RSO$_2$SR………………………………….……………………………………57
References to historical summary…………………………………………………………„58

Materials and methods

Instrumentation: NMR spectra were collected using a Bruker 500 MHz AVIIIHD system with a PRODIGY BBO probe installed (Bruker, Inc, Billerica, MA) (^{1}H: 500 MHz; ^{13}C: 125.7 MHz). All spectra were recorded in $CDCl_3$. Referencing was to $CHCl_3$ for ^{1}H NMR. The spectra were processed using Topspin 3.5 software (Bruker, Inc). DART high-resolution mass spectra of the synthesized compounds were acquired using a DART-SVP ion source (IonSense, Saugus, MA) coupled to a JEOL AccuTOF high resolution time-of-flight mass spectrometer (JEOL USA, Peabody, MA). Infrared spectra were collected using a PerkinElmer FT-IR spectrometer (PerkinElmer, Waltham, MA).

Materials: Benzaldehyde, *p*-tolyl disulfide, *m*-chloroperoxybenzoic acid (*m*-CPBA), and bromine were purchased from Sigma-Aldrich (St. Louis, MO). Ethyl acetate and sodium hydroxide (NaOH) were obtained from VWR Int. (Radnor, PA). Methanol and dichloromethane (DCM) were purchased from Pharmco (Brookfield, CT). 2,4,6-Trimethylthiophenol and 2,4,6-trimethylbenzene sulfinamide were acquired from AmBeed (Arlington Hts., IL). Benzene was purchased from TCI America (Portland, OR). Potassium hydroxide (KOH) was obtained from Acros Organics (Geel, Belgium). Hexane was acquired from Honeywell (Charlotte, NC). Mg_2SO_4 was purchased from EMD Chemicals Inc. (Gibbstown, NJ). *p*-Tolyl sulfinamide was purchased from Oakwood Chemical (Estill, SC). Deuterated chloroform was purchased from Cambridge Isotope Laboratories Inc. (Tewksbury, MA).

List of Abbreviations:

DCM = dichloromethane, *m*-CPBA - *m*-chloroperoxybenzoic acid, TLC – thin layer chromatography, DART-HRMS – direct analysis in real time – high-resolution mass spectrometry, XAS – x-ray absorption spectroscopy, DFT - density functional theory, SSRL - Stanford Synchrotron Radiation Lightsource, NMR – nuclear magnetic resonance, IR - infrared

Synthesis of disulfides

1,3,5-Trimethyl-2-[(2,4,6-trimethylphenyl)disulfanyl]benzene (mesityl disulfide)

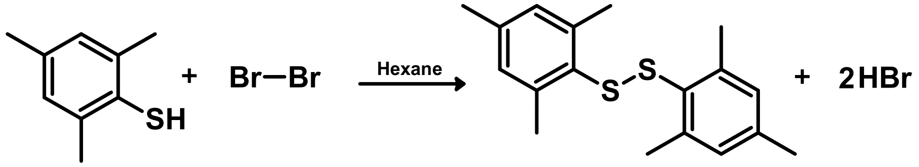

The disulfide was synthesized according to a published procedure, with some modifications.[1] To a stirred solution of 2,4,6-trimethylthiophenol (26.0 mmol, 4.0 g) in hexane (122.6 mmol, 16 mL) at room temperature, bromine (26.7 mmol, 0.69 mL) diluted in hexane (7.7 mmol, 1 mL) was added dropwise. The color of the final reaction was a pale yellow. Following evaporation of the solvent, the solid yellow residue was washed with ice-cold methanol and filtered under vacuum. The crystals were air dried. A total of 1.98 grams of mesityl disulfide was obtained as a pale-yellow crystalline powder. **Yield:** 50%.

Mp: 123-124 °C (corrected).[2,3]

IR (cm^{-1}): ν = 3021, 2984, 2948, 2914, 2847, 1598, 1434, 1370, 1294, 1030, 862, 852, 558

^{1}H-NMR (500 MHz, $CDCl_3$): δ = 6.87 (s, 4H), 2.29 (s, 6H), 2.24 (s, 12H)

^{13}C-NMR (125 MHz, $CDCl_3$): δ = 143.3, 139.3, 131.6, 128.9, 21.5, 21.2

HRMS (DART) *m/z*: $[M]^+$ Calcd for $C_{18}H_{22}S_2$ 302.1163; Found 302.1160.

Synthesis of thiosulfinates

1-Methyl-4-(4-methylphenyl)sulfinylsulfanylbenzene (*p*-tolyl thiosulfinate)

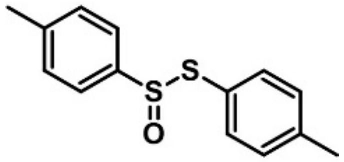

To a stirred solution containing *p*-tolyl disulfide (20.6 mmol, 5.1 g) in DCM (531.1 mmol, 34 mL) at -78 ºC, was added dropwise a solution of *m*-CPBA (20.6 mmol, 3.5 g) in DCM (531.1 mmol,

---

[1] Wu, X.; Rieke, R. D.; Zhu, L. Preparation of Disulfides by the Oxidation of Thiols Using Bromine. Syn. Commun. **1996**, *26* (1), 191–196. https://doi.org/10.1080/00397919608003879.

[2] The observed MP aligned with that reported in: Kawahara, K. Studies on Synthesis of Diaryl Disulfide. II. Journal of the Pharmaceutical Society of Japan 1957, 77 (9), 963–965. https://doi.org/10.1248/yakushi1947.77.9_963. (MP reported 123-124 °C)

[3] The MP observed in this work does not align with that reported in: Souto, J. A.; Lewis, W.; Stockman, R. A. Isolation of stable non cyclic 1,2-disulfoxides. Revisiting the thermolysis of S-aryl sulfinimines. *Chem. Commun*. **2014**, *50*, 12630-12632. https://doi.org/10.1039/C4CC05751A (MP reported 50-52°C)

34 mL). Upon completion of the addition, the mixture was removed from the cold bath and allowed to stir for 3 h at room temperature. An aqueous solution of 10% NaOH was then added, and the resulting mixture was extracted with DCM (4×). The DCM extracts were pooled and the resulting solution was dried (Na_2SO_4) and filtered. The solvent was then evaporated. The crude residue was subjected to preparative TLC (Analtech Preadsorbent Silica gel GF Taper, 20×20cm, 300-1700 micron, hexane:ethyl acetate 20:1). The band corresponding to *p*-tolyl thiosulfinate was scraped and suspended in ethyl acetate (1023.7 mmol, 100 mL). Filtration followed by evaporation of the solvent furnished 609 mg of *p*-tolyl thiosulfinate as a pale-yellow powder. **Yield:** 11%.

Mp: 86-87 °C (corrected).[4]

IR (cm^{-1}): ν = 1590, 1488, 3054, 2961, 2916, 2856, 1091, 806

^{1}H-NMR (500 MHz, $CDCl_3$): δ = 7.59 (d, *J* = 8.3 Hz, 2H), 7.46 (d, *J* = 8.0, Hz 2H), 7.33 (d, *J* = 7.9 Hz, 2H), 7.22 (d, *J* = 7.8 Hz, 2H), 2.44 (s, 3H), 2.41 (s, 3H)

^{13}C-NMR (125 MHz, $CDCl_3$): δ = 142.2, 141.0, 140.8, 135.4, 129.7, 126.2, 124.3, 21.5, 21.4

HRMS (DART) *m/z*: $[M + H]^+$ Calcd for $C_{14}H_{15}OS_2$ 263.0564; Found 263.0550

<u>1,3,5-Trimethyl-2-[(2,4,6-trimethylphenyl)sulfinylsulfanylbenzene</u> (mesityl thiosulfinate)

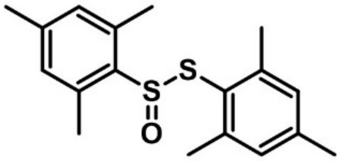

To a stirred solution containing mesityl disulfide (2.7 mmol, 0.8 g) in DCM (313.4 mmol, 20 mL) at -78 ºC was added dropwise a solution of *m*-CPBA (3.4 mmol, 0.6 g) in DCM (468.6 mmol, 30 mL). Upon completion of the addition, the mixture was removed from the cold bath and allowed to stir for 3 h at room temperature. An aqueous solution of 10% NaOH was then added, and the resulting mixture was extracted with DCM (4×). The DCM extracts were pooled and the resulting solution was dried (Na_2SO_4) and filtered. The solvent was then evaporated. The crude residue was subjected to preparative TLC (Analtech Preadsorbent Silica gel GF Taper, 20×20cm, 300-1700 micron, hexane:ethyl acetate 20:1). The band corresponding to the mesityl thiosulfinate was

[4] The MP observed here was similar to that reported in: McNeil, N. M. R.; McDonnell, C.; Hambrook, M.; Back, T. G. Oxidation of Disulfides to Thiosulfinates with Hydrogen Peroxide and a Cyclic Seleninate Ester Catalyst. *Molecules* **2015**, *20*, 10748–1076;. https://doi.org/10.3390/molecules200610748. (MP reported 85-86 °C)

scraped and suspended in ethyl acetate (1023.7 mmol, 100 mL). Filtration followed by evaporation of the solvent furnished 291 mg of mesityl thiosulfinate as a pale-yellow powder. **Yield:** 34%.

Mp: 115-116 °C (corrected).

IR (cm^{-1}): ν = 3018, 2963, 2920, 2851, 1599, 1452, 1099

^{1}H-NMR (500 MHz, $CDCl_3$): δ = 7.04 (s, 2H), 6.93 (s, 2H), 2.59 (s, 6H), 2.34 (s, 3H), 2.33 (s, 3H), 2.20 (s, 6H).

^{13}C-NMR (125 MHz, $CDCl_3$): δ = 144.7, 141.7, 140.7, 137.7, 131.9, 129.6, 129.5, 126.0, 22.7, 21.2, 21.2, 19.2.

HRMS (DART) *m/z*: $[M + H]^+$ Calcd for $C_{18}H_{23}OS_2$ 319.1190; Found 319.1188

Synthesis of sulfinimines

N-Benzylidene-4-methylbenzenesulfinamide (benzylidene-*p*-toluenesulfinimine)

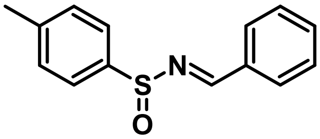

The procedure was based on that reported by Ardej-Jakubisiak *et al.*, 2007, with some modifications.[5] To a solution of *p*-tolyl sulfinamide (12.9 mmol, 2.0 g) in methanol (1569.7 mmol, 63.5 mL) was added KOH (12.9 mmol, 0.7 g). The mixture was stirred for 15 min to dissolve the KOH, and benzaldehyde (19.3 mmol, 2.0 mL) was then added. The reaction mixture was stirred at room temperature for 16 h. The methanol was evaporated and the residue was washed with deionized water (5539.4 mmol, 100 mL). The product was air dried to furnish 2.44 g of *p*-tolyl sulfinimine as a white crystalline powder. **Yield:** 78%.

Mp: 71-72 °C (corrected).[6]

IR (cm^{-1}): ν = 1603, 1571, 1449, 1099, 687, 486, 455

^{1}H-NMR (500 MHz, $CDCl_3$): δ = 8.78 (s, 1H), 7.88 (d, 2H), 7.67 (d, 2H), 7.53 (m, 1H), 7.47 (t, 2H), 7.34 (d, 2H), 2.42 (s, 3H)

---

[5] Ardej-Jakubisiak, M.; Kawęcki, R.; Aneta Świetlińska. A Facile Synthesis of *N*-Sulfinylaldimines. *Tetrahedron-Asymmetry* **2007**, *18*, 2507–2509; https://doi.org/10.1016/j.tetasy.2007.10.015.

[6] Davis, F. A.; Friedman, A. J.; Nadir, U. K. Chemistry of the Sulfur-Nitrogen Bond. 14. Arenesulfenic Acids from N-Alkylidenearenesulfinamides (Sulfinimines). *J. Am. Chem. Soc.* **1978**, *100*, 2844–2852; https://doi.org/10.1021/ja00477a045. (MP reported 73-75 °C)

^{13}C-NMR (125 MHz, CDCl$_3$): δ = 160.7, 141.8, 141.8, 133.9, 132.6, 129.9, 129.6, 128.9, 124.8, 21.4

HRMS (DART) *m/z*: [M + H]$^+$ Calcd for $C_{14}H_{14}NOS$ 244.0796; Found 244.0775

N-Benzylidene-2,4,6-trimethylbenzenesulfinamide (benzylidene-mesitylsulfinimine)

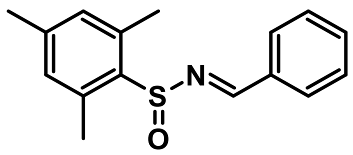

The procedure was based on that reported by Ardej-Jakubisiak *et al.*, 2007, with some modifications.[5] To a solution of 2,4,6-trimethylbenzenesulfinamide (4.1 mmol, 0.7 g) in methanol (519.1 mmol, 21.0 mL) was added KOH (4.1 mmol, 0.2 g). The mixture was stirred for 15 min to dissolve the KOH, and benzaldehyde (4.0 mmol, 0.6 mL) was then added. The reaction mixture was stirred at room temperature for 3 h. The methanol was evaporated and the residue was washed with deionized water (5539.4 mmol, 100 mL). The product was air dried to furnish 1.00 g of *N*-benzylidene-2,4,6-trimethylbenzene sulfinimine as a white crystalline powder. **Yield:** 91%

Mp: 109-110 °C (corrected).

IR (cm^{-1}): ν = 1601, 1571, 1450, 1090, 441

^{1}H-NMR (500 MHz, CDCl$_3$): δ = 8.86 (s, 1H), 7.87 (d, 2H), 7.54 (t, 1H), 7.48 (t, 2H), 6.89 (s, 2H), 2.53 (s, 6H), 2.31 (s, 3H)

^{13}C-NMR (125 MHz, CDCl$_3$): δ = 161.7, 141.7, 138.5, 135.4, 133.9, 132.5, 130.9, 129.5, 128.9, 21.1, 18.9

HRMS (DART) *m/z*: [M + H]$^+$ Calcd for $C_{16}H_{18}NOS$ 272.1109; Found 272.1112

Thermolysis reactions

Thermolysis of *N*-benzylidene-4-methylbenzenesulfinamide

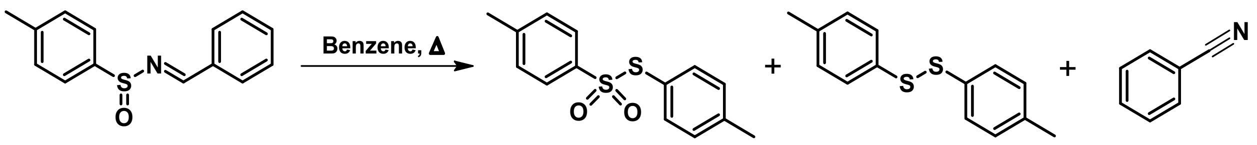

A solution of the *p*-tolyl thiosulfinimine (5.13 mmol, 1.2 g) in benzene (841.1 mmol, 75.0 mL) was refluxed for 15 h. The solvent was then evaporated and the residue was resuspended in a solution of hexane:ethyl acetate (5:1) (9 mL). Following gravity filtration, the filtrate mixture was subjected to preparative TLC (Analtech Preadsorbent Silica gel GF Taper, 20×20 cm, 300-1700 micron), hexane:ethyl acetate 20:1). Four bands were observed, corresponding to benzonitrile, *p*-tolyl disulfide, unreacted *p*-tolylsulfinimine, and a compound with a high-resolution mass of 279.0514 (corresponding to [$C_{14}H_{14}O_2S_2$ + H^+]). The band corresponding to this compound was scraped and the silica gel was suspended in ethyl acetate (100 mL). The solution was then vacuum filtered. Evaporation of the solvent furnished 108.12 mg of the compound as a white powder (**Yield:** 8%). In principle, this molecule could have represented *p*-tolyl thiosulfonate or *p*-tolyl disulfoxide. The latter would be expected to exhibit chirality at both sulfur atoms, resulting in the observation of a mixture of diastereomers (a pair of enantiomers and a *meso* compound—i.e., *R*,*R*; *S*,*S*; and *S*,*R*). To determine whether this product with the formula $C_{14}H_{14}O_2S_2$ was comprised of a mixture of stereoisomers, the crystals were subjected to chiral TLC analysis. Only a single band was ever observed, indicating either that the formula corresponded to the achiral thiosulfonate or the achiral *meso* disulfoxide. However, determination of its crystal structure revealed it to be *p*-tolyl thiosulfonate.

Mp: 77 – 78 °C (corrected).[7]

IR (cm^{-1}): ν = 3035, 2958, 2915, 2850, 1590, 1488, 1322, 1135, 805

^{1}H-NMR (500 MHz, $CDCl_3$): δ = 7.49 (d, *J* = 8.3, 2H), 7.28-7.23 (m, 4H), 7.17 (d, *J* = 7.9, 2H), 2.45 (s, 3H), 2.40 (s, 3H)

^{13}C-NMR (125 MHz, $CDCl_3$): δ = 144.6, 142.1, 140.5, 136.5, 130.2, 129.4, 127.6, 124.6, 21.7, 21.5

HRMS (DART) *m/z*: $[M + H]^+$ Calcd for $C_{14}H_{15}O_2S_2$ 279.0513; Found 279.0514

[7] The observed MP aligned with that reported in: Carson, J.; Wong, F. Notes- The Reactions of Thiosulfonates and Thiosulfinates with 1-Fluoro-2,4-Dinitrobenzene. *J. Org. Chem.* **1961**, 26, 3028–3030. https://doi.org/10.1021/jo01066a637. (MP reported 77-78 °C)

Thermolysis of *N*-benzylidene-2,4,6-trimethylbenzenesulfinamide

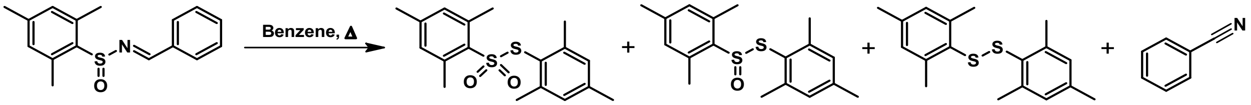

A solution of the 2,4,6-trimethylbenzenesulfinimine (2.60 mmol, 0.7 g) in benzene (459.8 mmol, 41.0 mL) was refluxed for 15 h. The solvent was evaporated and the residue was resuspended in ethyl acetate (102.4 mmol, 10 mL). Following gravity filtration, the mixture was subjected to preparative TLC (Analtech Preadsorbent Silica gel GF Taper, 20×20 cm, 300-1700 micron, hexane:ethyl acetate 20:1). Five bands corresponding to the following compounds were observed: unreacted starting material, benzonitrile, mesityl disulfide, mesityl thiosulfinate and mesityl thiosulfonate. The band corresponding to 2,4,6-trimethylbenzene thiosulfonate was scraped and the silica gel was resuspended in ethyl acetate (1023.7 mmol, 100 mL). The solution was then vacuum filtered. Evaporation of the solvent from the filtrate furnished 97.76 mg of 2,4,6-trimethylbenzene thiosulfonate as a white powder. **Yield:** 11%.

Mp: 135-136 °C (corrected)[8]

IR (cm^{-1}): ν = 3021, 2968, 2935, 2918, 2853, 1598, 1455, 1318, 1136, 1031, 851, 587, 511

^{1}H-NMR (500 MHz, $CDCl_3$): δ = 6.92 (s, 2H), 6.91 (s, 2H), 2.38 (s, 6H), 2.32 (s, 3H), 2.30 (s, 3H), 2.18 (s, 6H)

^{13}C-NMR (125 MHz, $CDCl_3$): δ = 145.4, 143.4, 141.8, 139.8, 139.5, 131.9, 129.5, 123.4, 22.7, 21.5, 21.2, 21.1

HRMS (DART) *m/z*: $[M + H]^+$ Calcd for $C_{18}H_{23}S_2O_2$ 335.1139; Found 335.1130

Synthesis of thiosulfonates from thiosulfinates

A solution of the *p*-tolyl thiosulfinate (1.145 mmol, 0.3 g) in benzene (235.5 mmol, 21.0 mL) was refluxed for 15 hours. The solvent was evaporated and the residue, dissolved in ethyl acetate, was purified by preparative TLC (Analtech Preadsorbent Silica gel GF Taper, 20×20 cm, 300-1700 micron, hexane:ethyl acetate 20:1). Seven bands were observed, three of which were confirmed to

---

[8] This MP aligns with that reported in: Palumbo, G.; Caputo, R. A Facile Way to Thiosulfonic S-Esters. *Synthesis* **1981**, *1981*, 888–890; https://doi.org/10.1055/s-1981-29634. (MP reported 133-134 °C) and is different from that reported in: Souto, J. A.; Lewis, W.; Stockman, R. A. Isolation of stable non cyclic 1,2-disulfoxides. Revisiting the thermolysis of *S*-aryl sulfinimines. *Chem. Commun*. **2014**, 50, 12630-12632; https://doi.org/10.1039/C4CC05751A (MP reported 79 – 81 °C).

correspond to: *p*-tolyl disulfide, *p*-tolyl thiosulfonate, and unreacted *p*-tolyl thiosulfinate. The other four compounds were present in trace amounts whose quantities were too low to permit further structural characterization experiments. The band corresponding to *p*-tolyl thiosulfonate was scraped and the silica gel was resuspended in ethyl acetate (100 mL). The solution was vacuum filtered. Evaporation of the solvent furnished 44.02 mg of *p*-tolyl thiosulfonate as a white powder. **Yield:** 28% yield.

Infrared spectra

<u>*N*-Benzylidene-4-methylbenzenesulfinamide</u> (benzylidene-*p*-toluenesulfinimine)

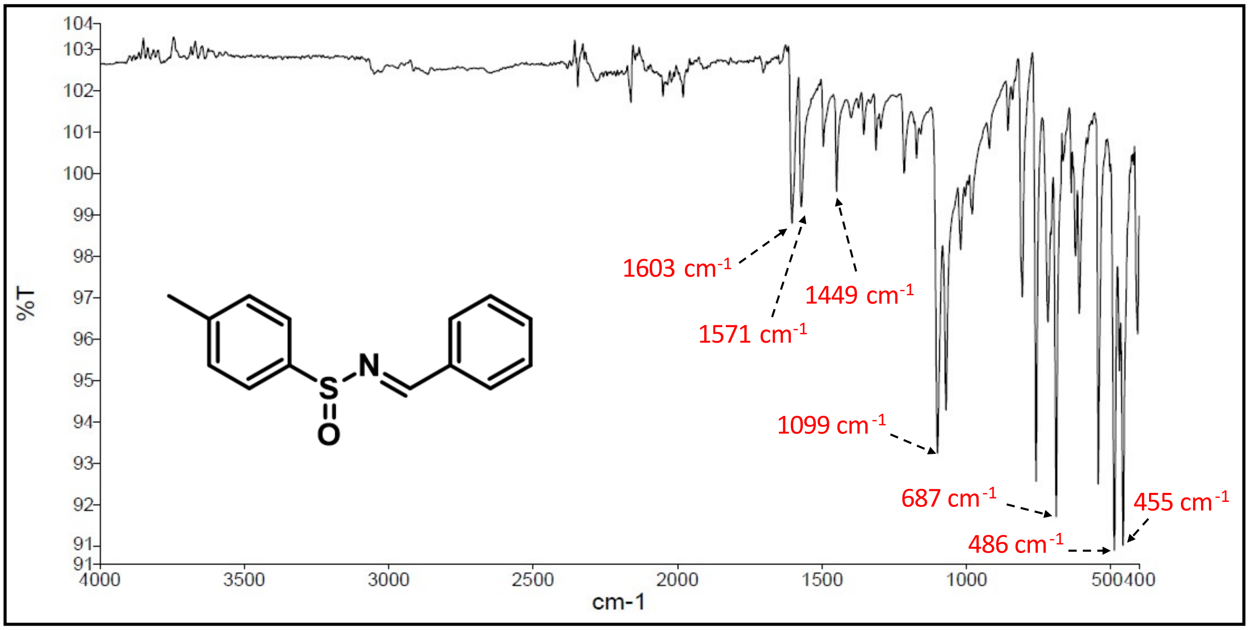

1-Methyl-4-(4-methylphenyl)sulfinylsulfanylbenzene (*p*-tolyl thiosulfinate)

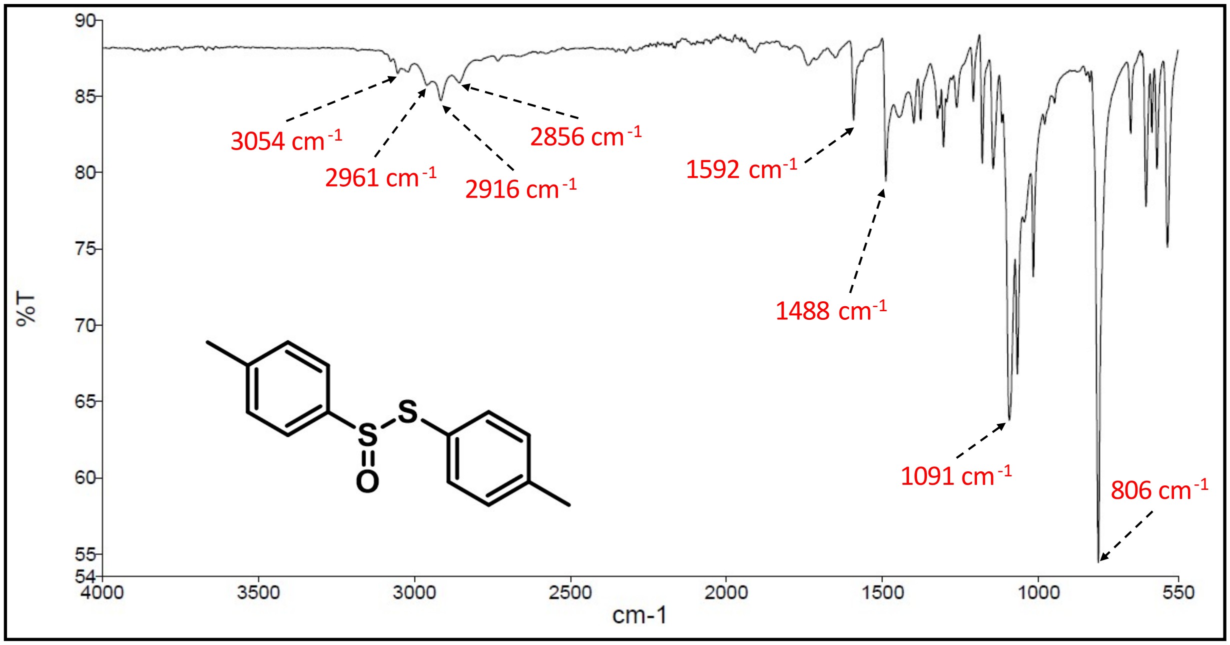

1-Methyl-4-(4-methylphenyl)sulfonylsulfanylbenzene (*p*-tolyl thiosulfonate)

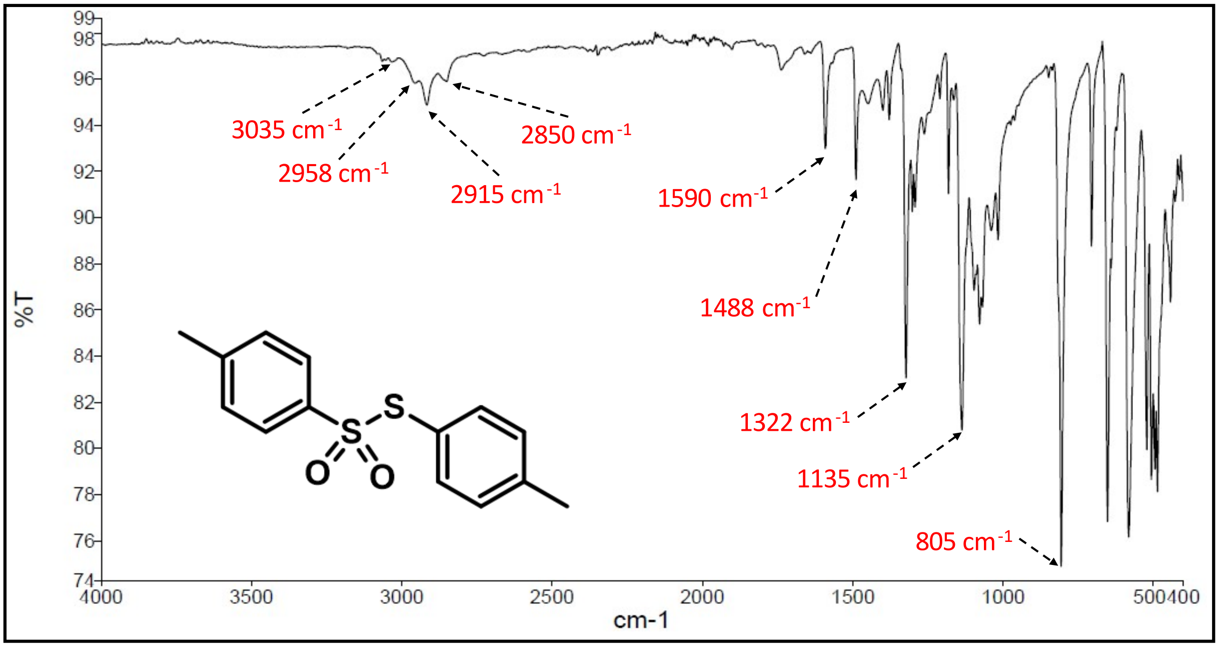

1,3,5-Trimethyl-2-[(2,4,6-trimethylphenyl)disulfanyl]benzene (mesityl disulfide)

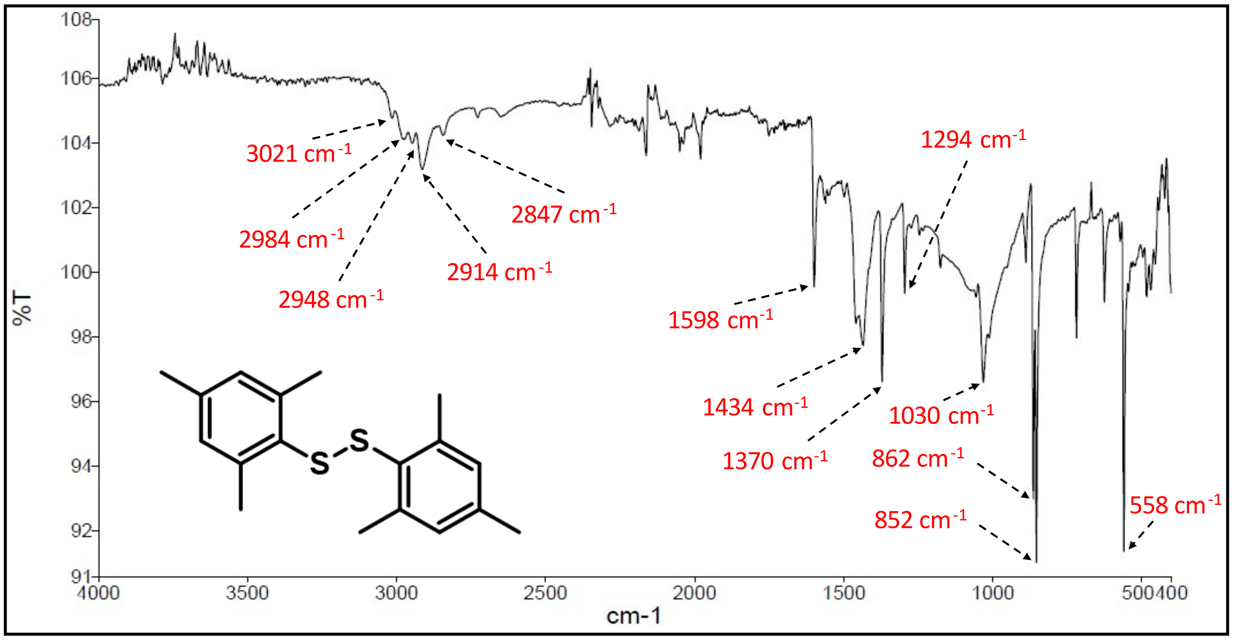

N-Benzylidene-2,4,6-trimethylbenzenesulfinamide (benzylidene-mesitylsulfinimine)

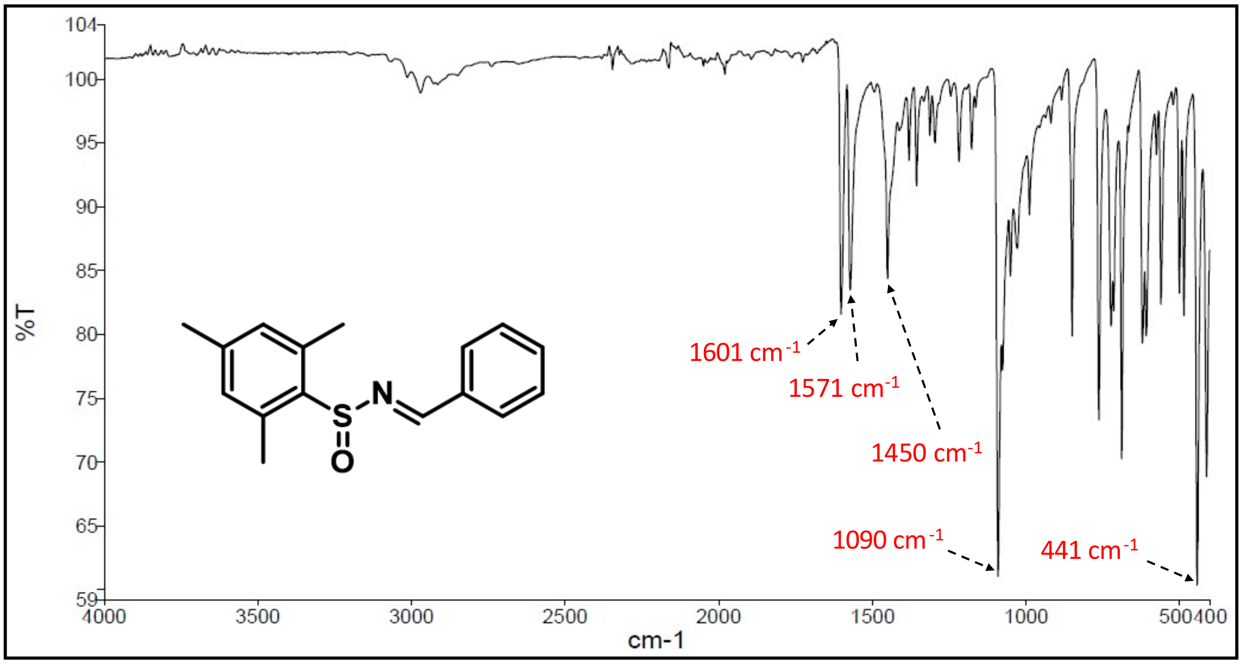

1,3,5-Trimethyl-2-[(2,4,6-trimethylphenyl)sulfinylsulfanylbenzene (mesityl thiosulfinate)

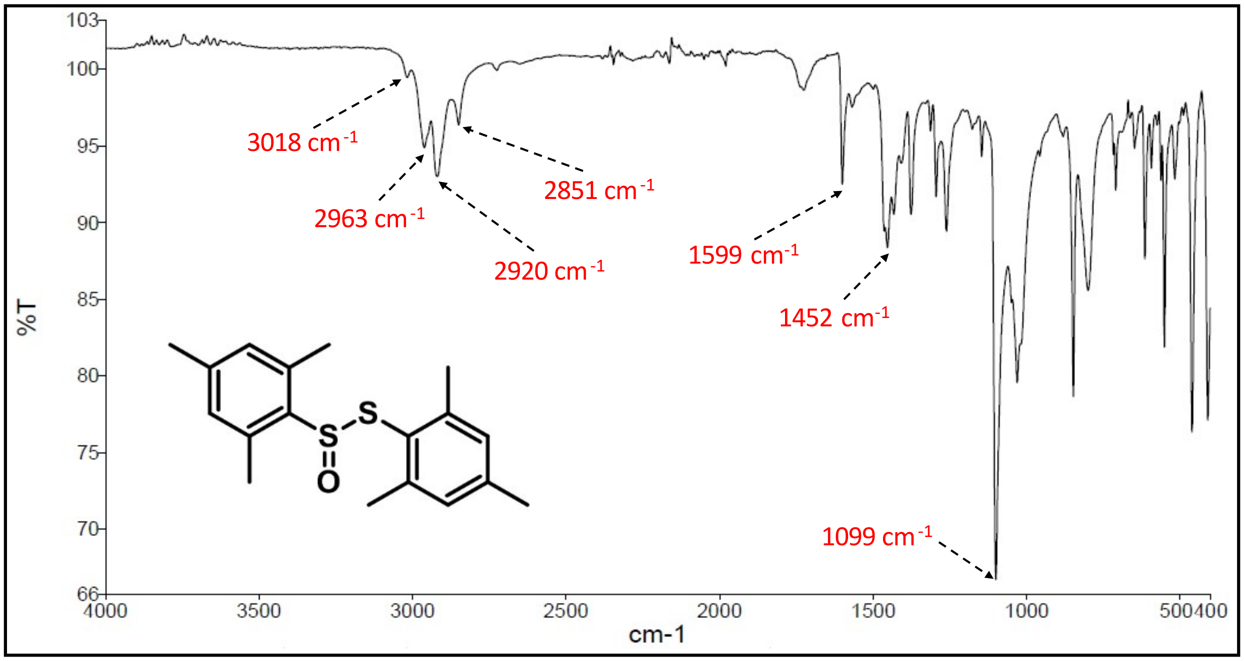

1,3,5-Trimethyl-2-(2,4,6-trimethylphenyl)sulfonylsulfanylbenzene (mesityl thiosulfonate)

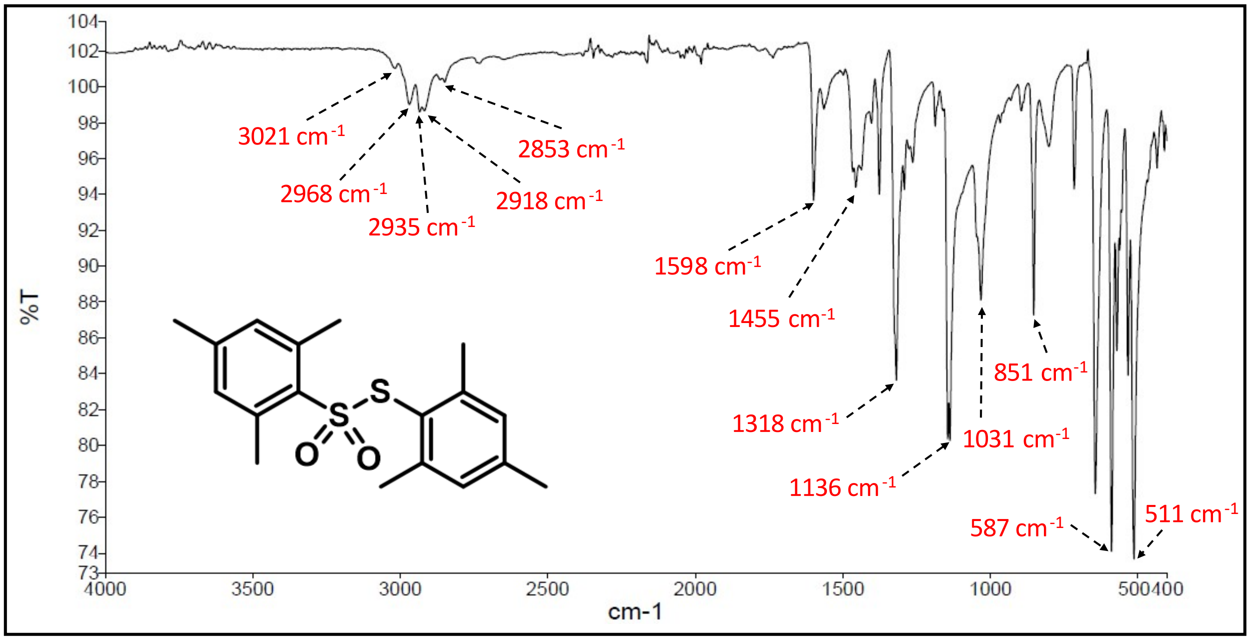

^{1}H-NMR and ^{13}C-NMR spectra

<u>*N*-Benzylidene-4-methylbenzenesulfinamide</u> (benzylidene-*p*-toluenesulfinimine)

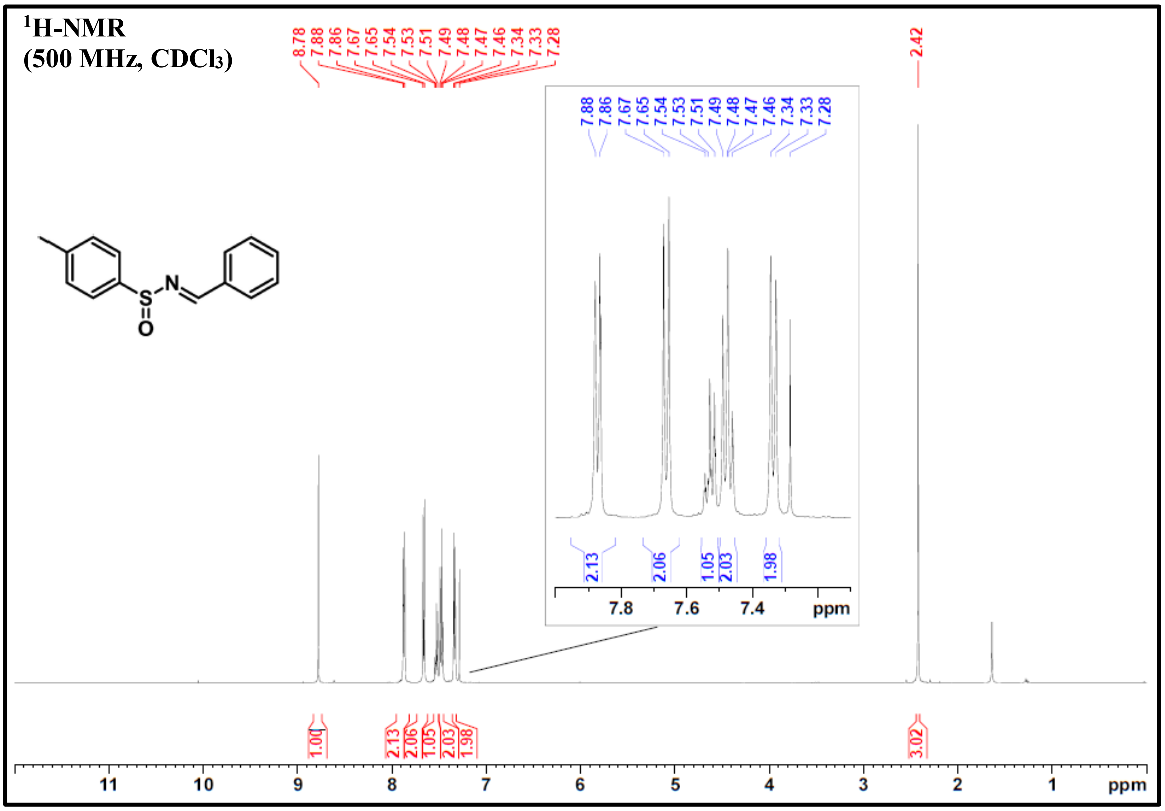

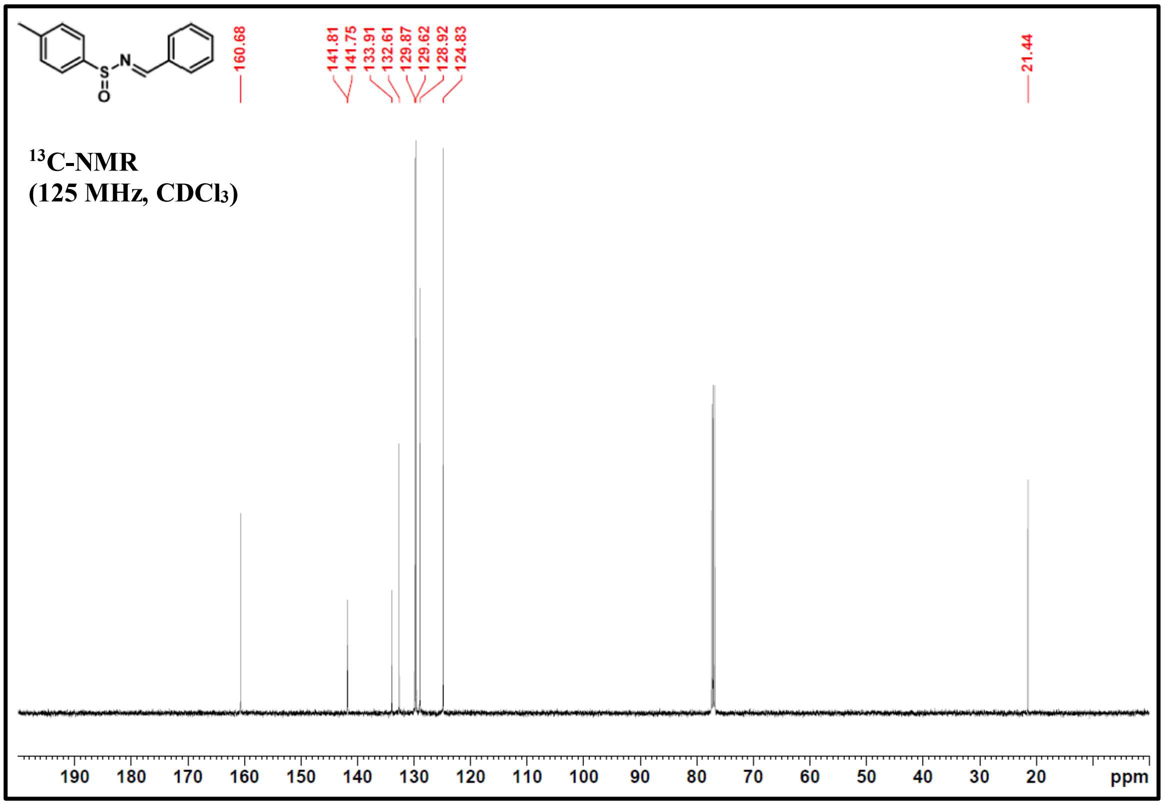

p-1-Methyl-4-(4-methylphenyl)sulfinylsulfanylbenzene (*p*-tolyl thiosulfinate)

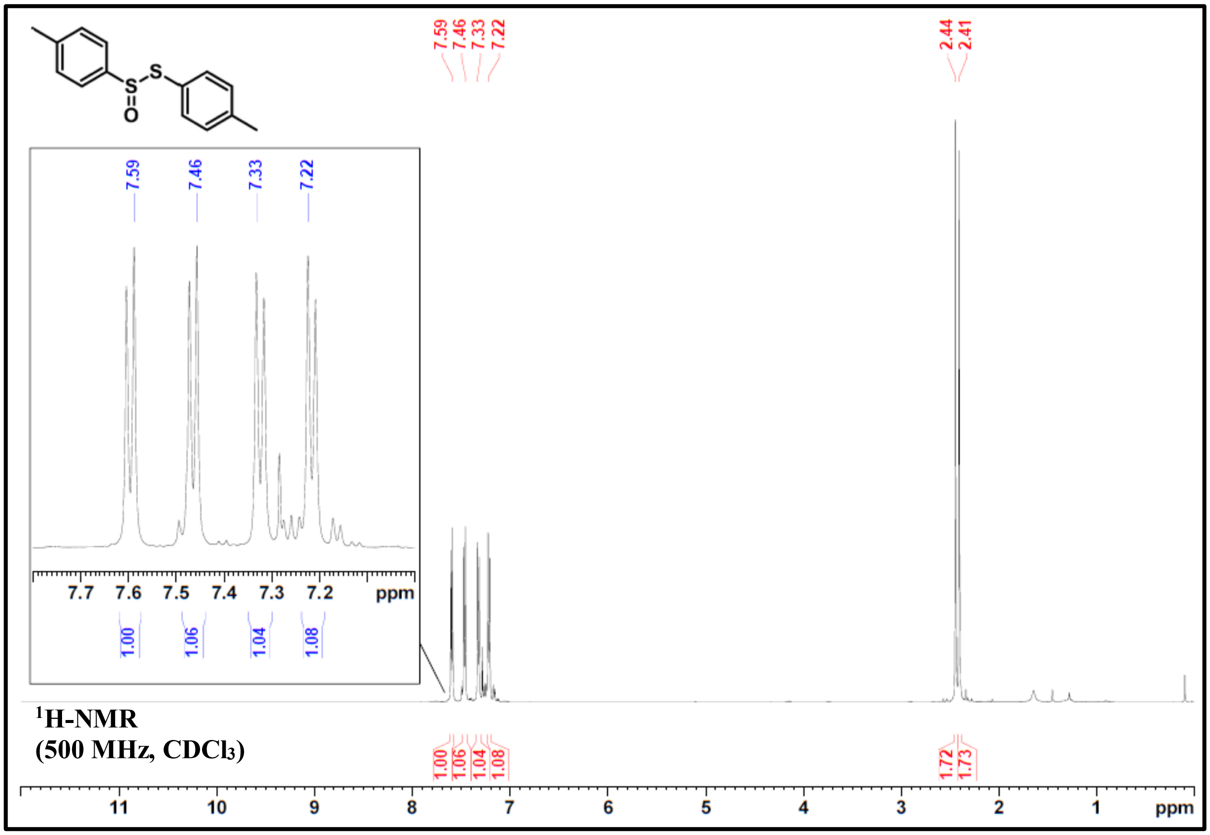

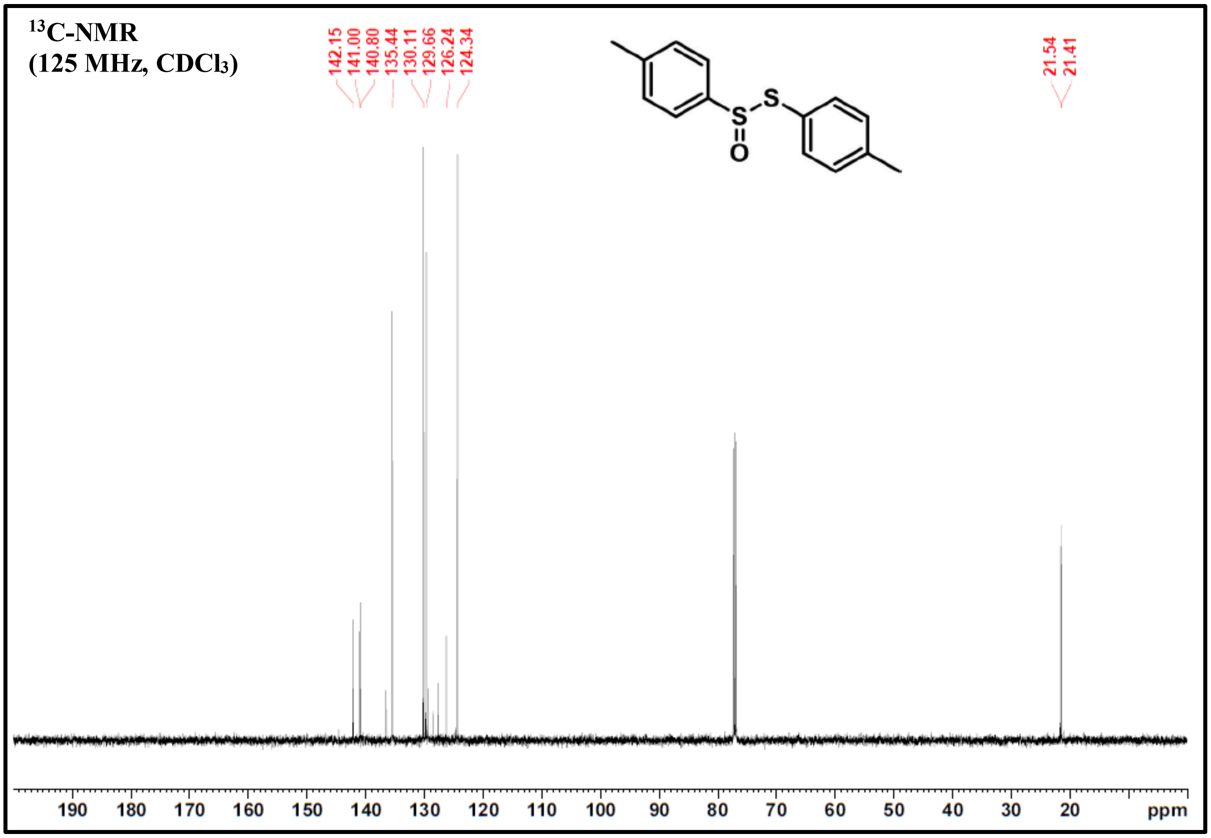

1-Methyl-4-(4-methylphenyl)sulfonylsulfanylbenzene (*p*-tolyl thiosulfonate)

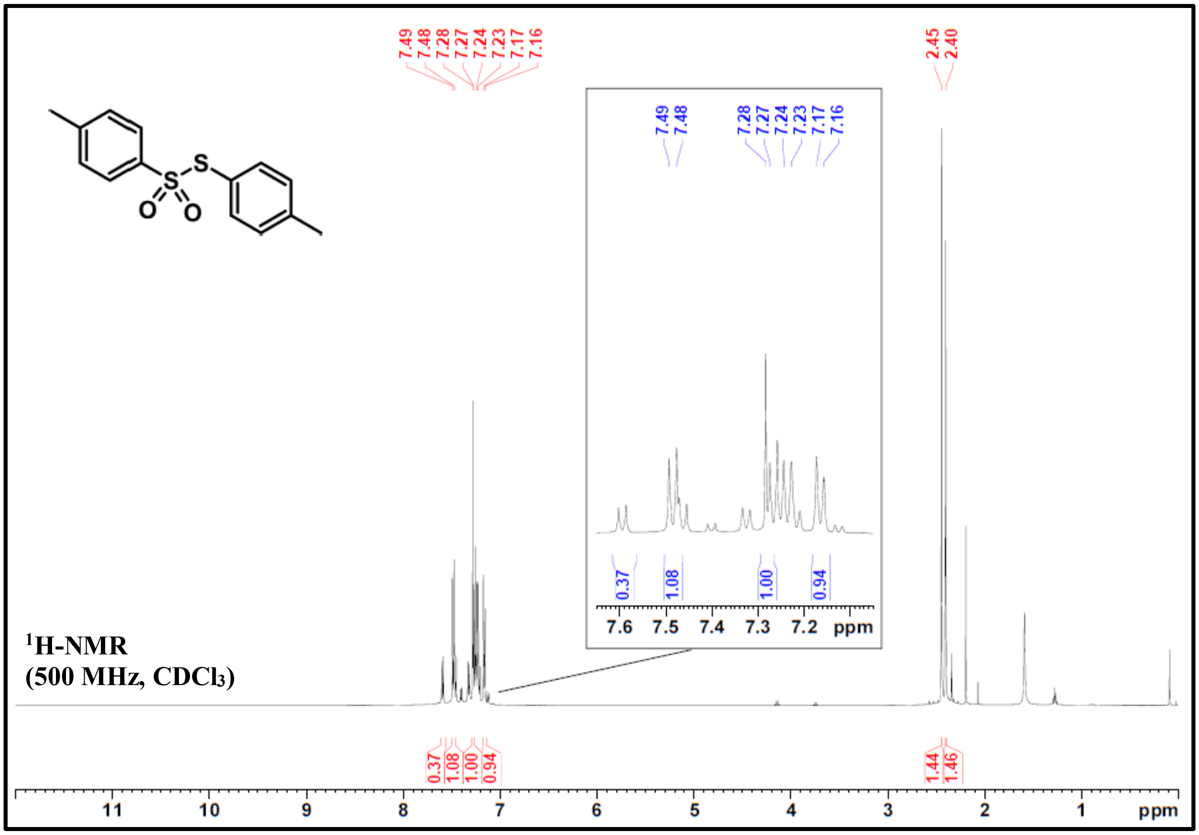

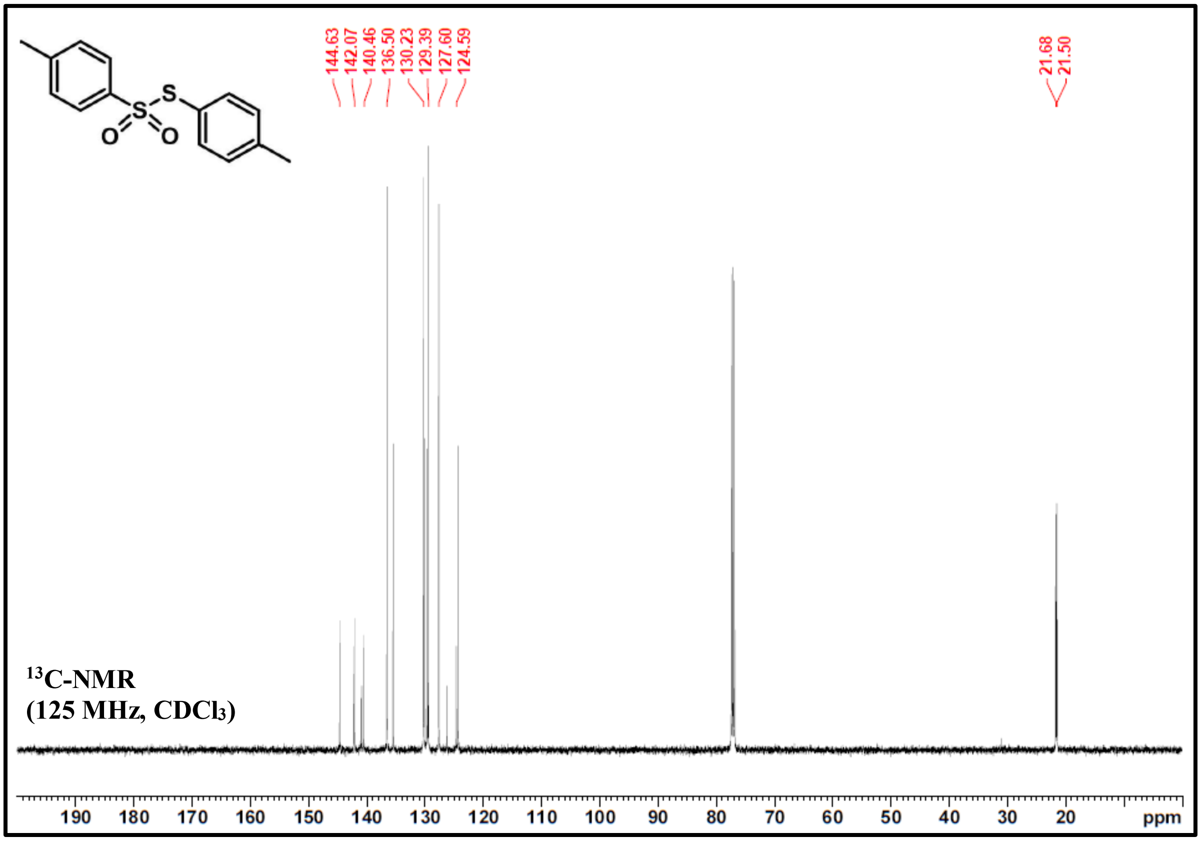

1,3,5-Trimethyl-2-[(2,4,6-trimethylphenyl)disulfanyl]benzene (mesityl disulfide)

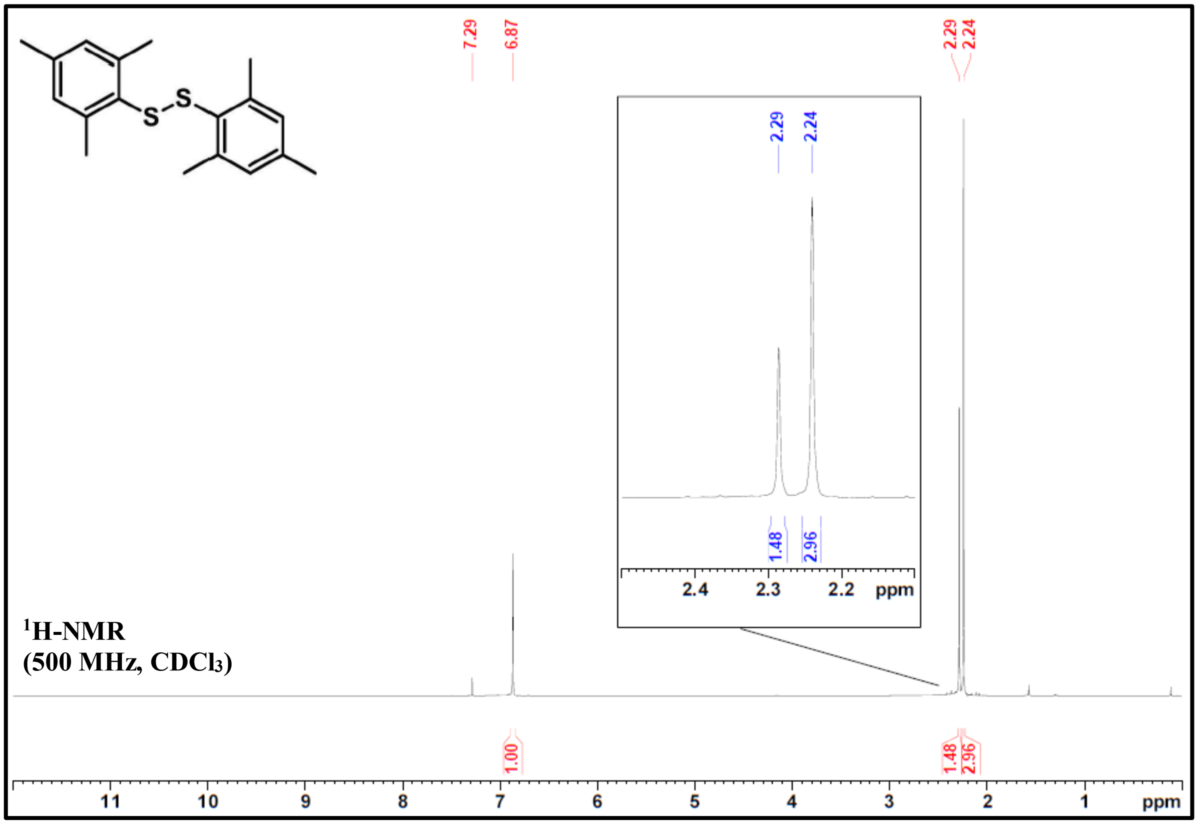

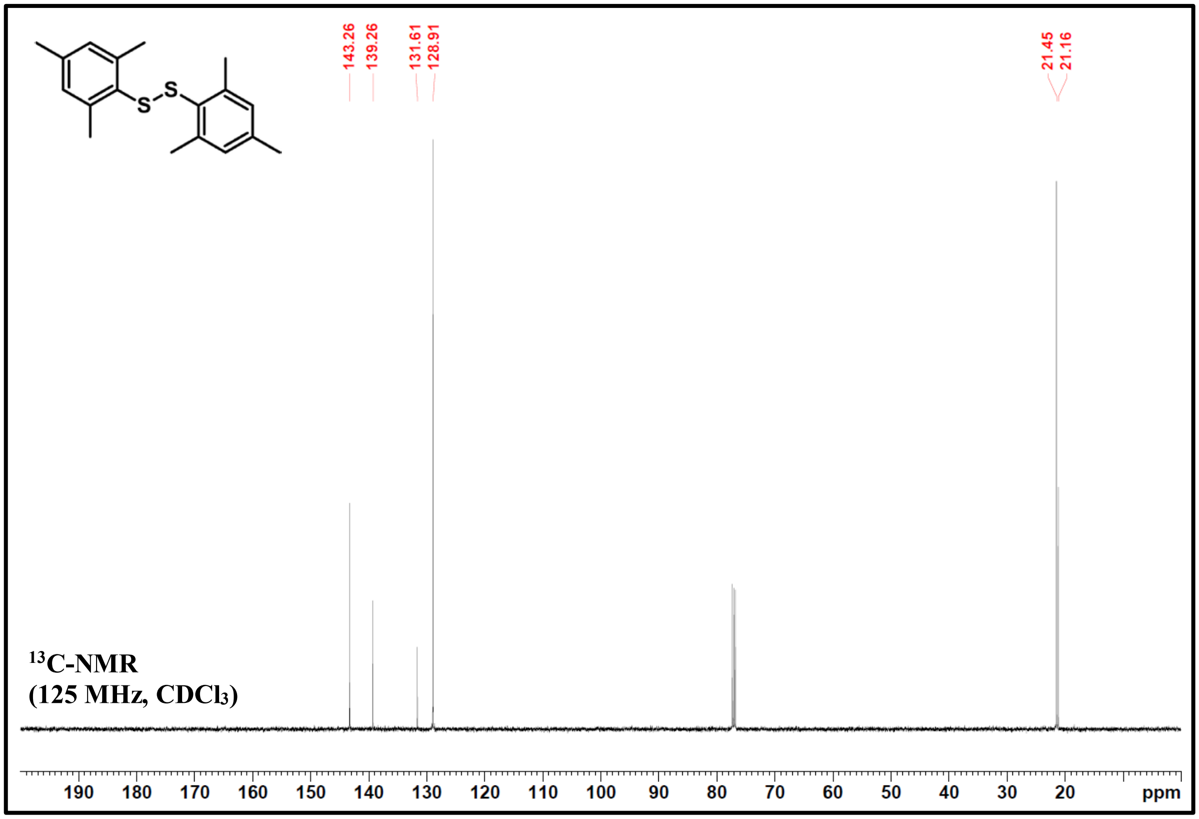

N-Benzylidene-2,4,6-trimethylbenzenesulfinamide (benzylidene-mesitylsulfinimine)

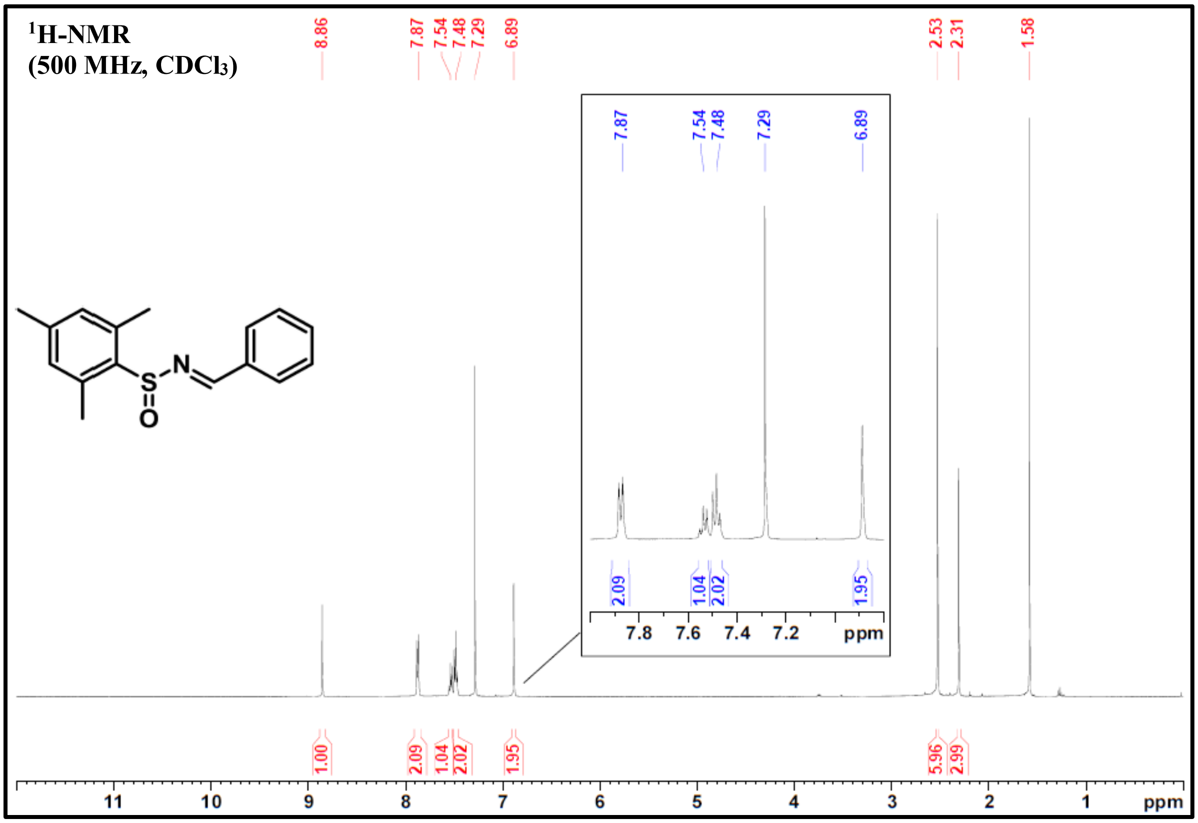

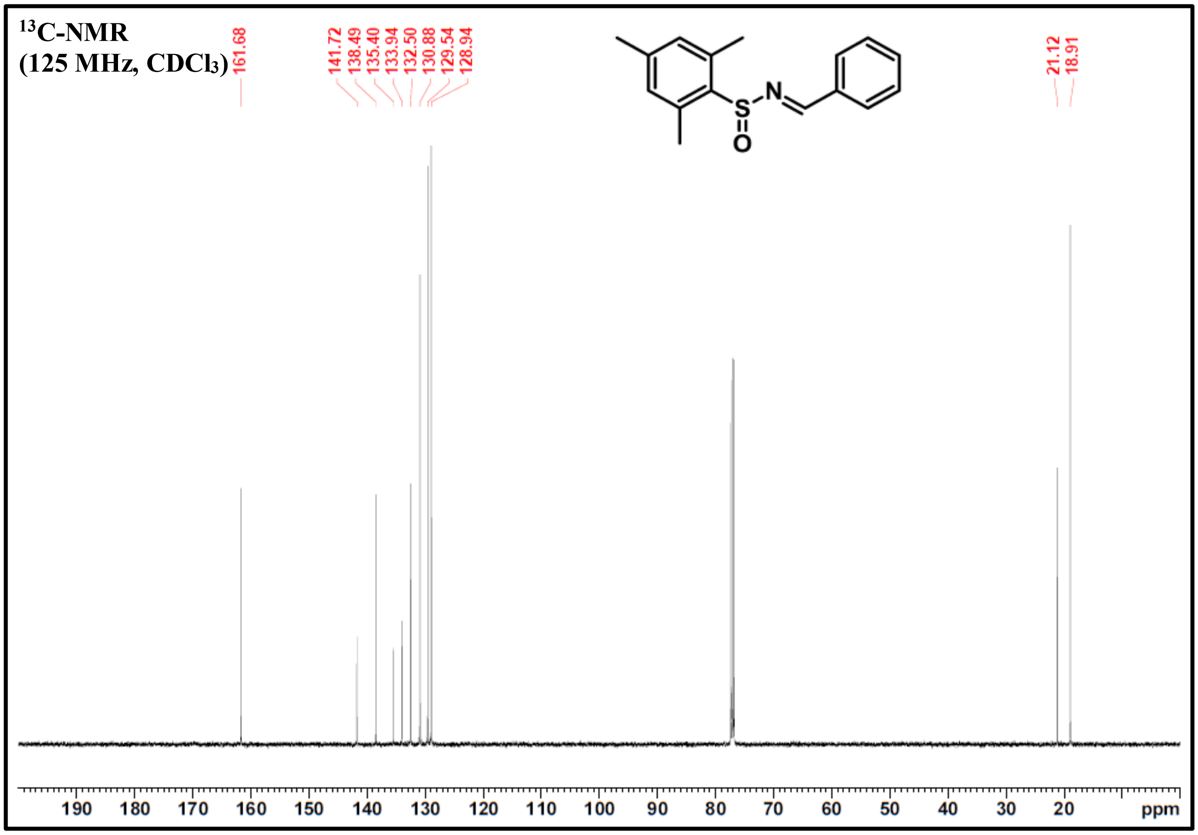

1,3,5-Trimethyl-2-[(2,4,6-trimethylphenyl)sulfinylsulfanylbenzene (mesityl thiosulfinate)

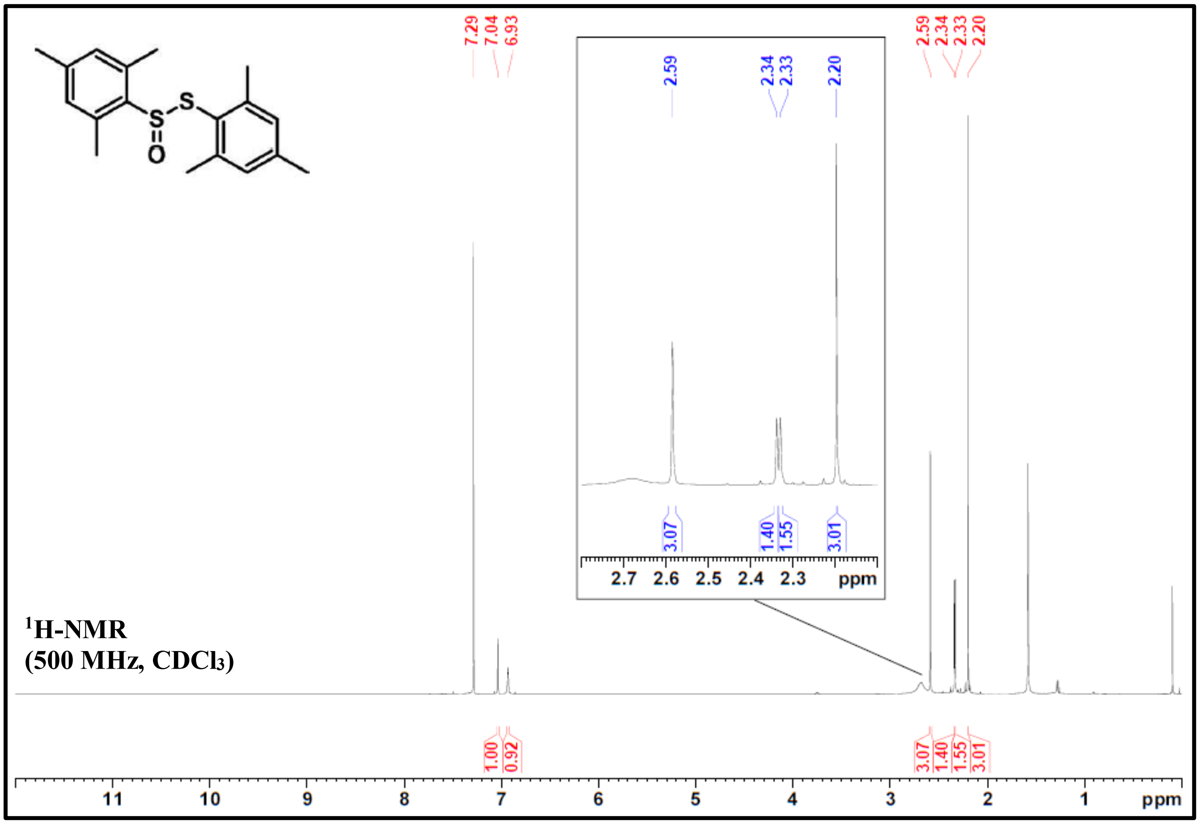

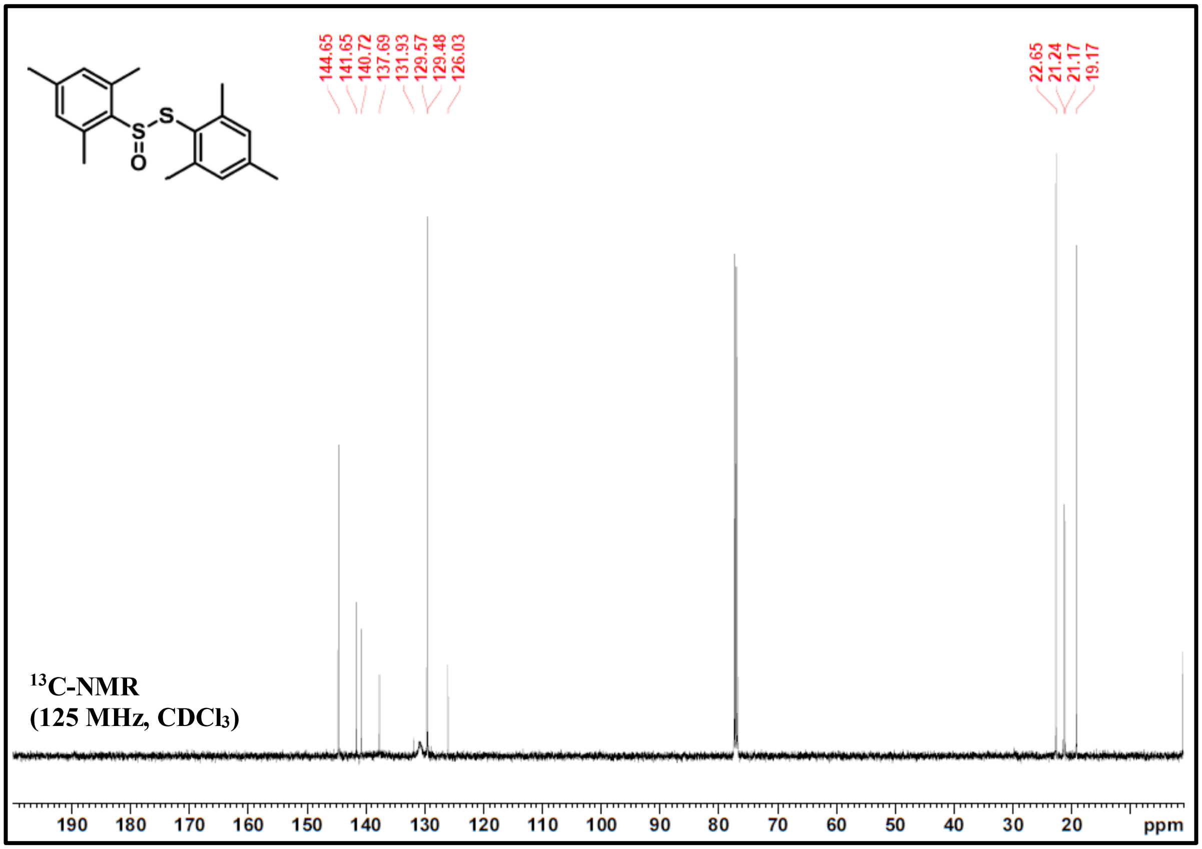

1,3,5-Trimethyl-2-(2,4,6-trimethylphenyl)sulfonylsulfanylbenzene (mesityl thiosulfonate)

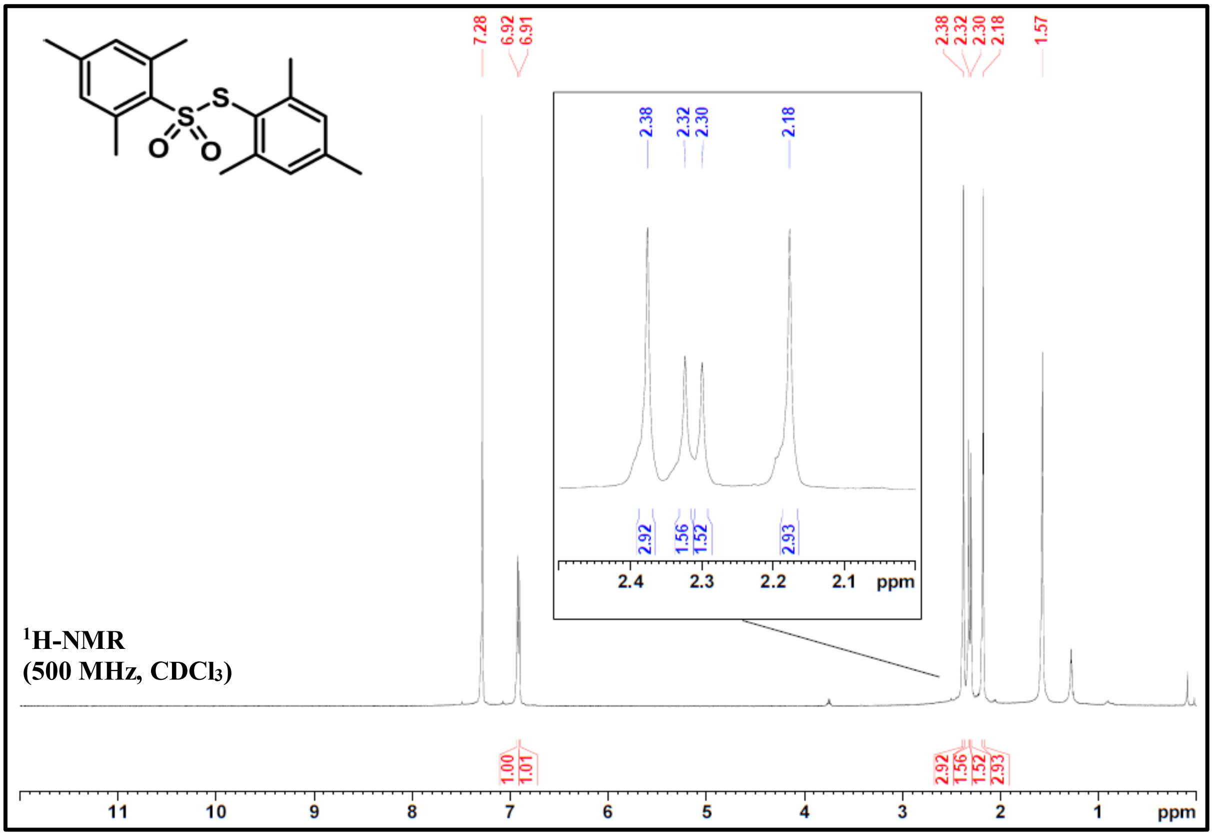

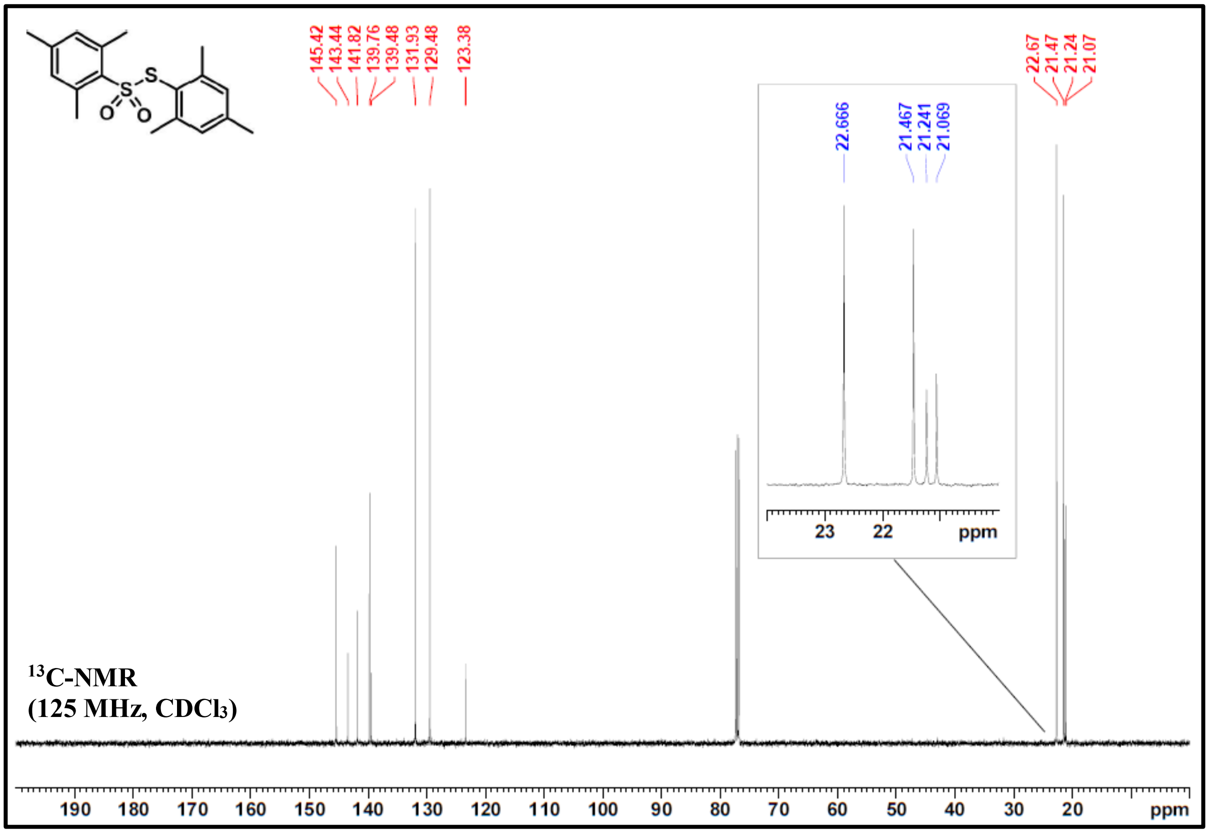

Crystal structure solution and refinement

Data collections of di-tolyl thiosulfonate and di-mesityl thiosulfonate were performed at 100(2) K on a Rigaku XtaLAB Synergy-S X-ray diffractometer equipped with a HyPix-6000HE hybrid photon counting (HPC) detector and a microfocus Cu-Kα radiation (λ = 1.54178 Å). The data collection strategy to ensure completeness and the desired redundancy were determined using CrysAlis^{Pro}.[9] Data processing was performed using CrysAlis^{Pro}. Empirical absorption correction was applied using the SCALE3 ABSPACK scaling algorithm.[10] The structures were solved by SHELXT (version 2018/2)[11] and refined by full-matrix least-squares procedures using the Bruker SHELXTL (version 2019/3)[12] software package through the OLEX2 graphical interface.[13] All non-hydrogen atoms, including those in disordered parts, were refined anisotropically. Hydrogen atoms were included in idealized positions for structure factor calculations with $U_{iso}(H) = 1.2\ U_{eq}(C)$ and $U_{iso}(H) = 1.5\ U_{eq}(C)$ for methyl groups. In the structure model of di-tolyl thiosulfonate, the hydrogen atoms of two methyl groups were found to be disordered and were modeled with two orientations rotated from each other by 60 degrees with all their occupation factors fixed at 0.5. In the structure model of di-mesityl thiosulfonate, two oxygens were found to be disordered over two sulfurs and were modeled with two orientations. Their relative occupancy factors were refined to 0.893(3): 0.107(3). Further crystal and data collection details are listed in Table S1.

[9] Rigaku Corporation. Rigaku Oxford Diffraction, CrysAlisPro Software System, Version 171.43.112a. **2023**.
[10] Rigaku Oxford Diffraction. SCALE3 ABSPACK; A Rigaku Oxford Diffraction Program (1.0.11, Gui:1.0.7) (C). **2005**.
[11] G. M. Sheldrick, *Acta Crystallogr.* **2015**, *A71*, 3-8.
[12] G. M. Sheldrick, *Acta Crystallogr.* **2015**, *C71*, 3-8.
[13] O. V. Dolomanov, L. J. Bourhis, R. J. Gildea, J. A. K. Howard, H. Puschmann, *J. Appl. Crystallogr.* **2009**, *42*, 339-341.

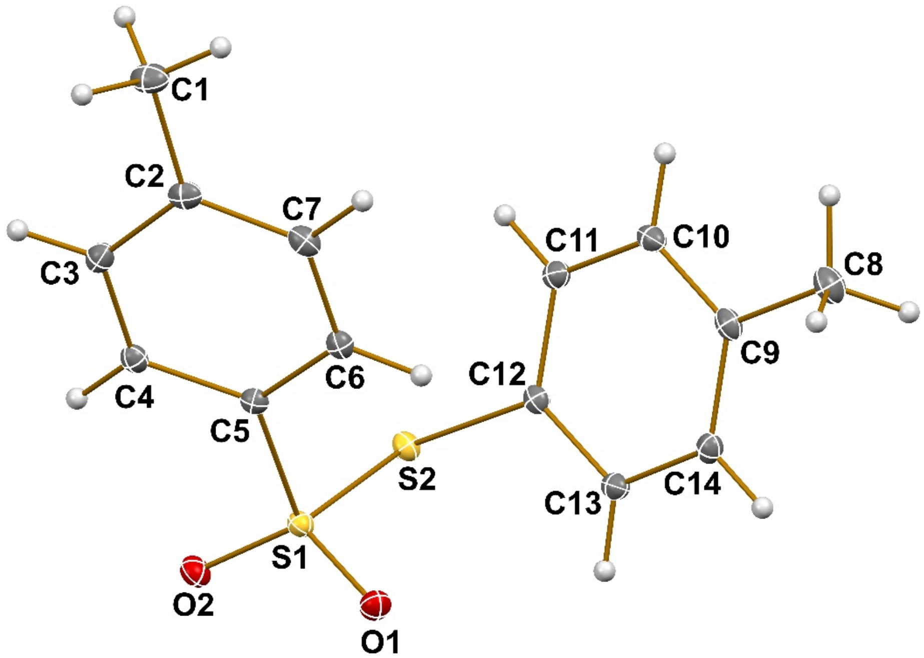

Figure S1. The molecular structure of di-tolyl thiosulfonate at 100(2) K. Atoms are represented by thermal ellipsoids at the 40% probability level. H atoms are shown as spheres of arbitrary radius.

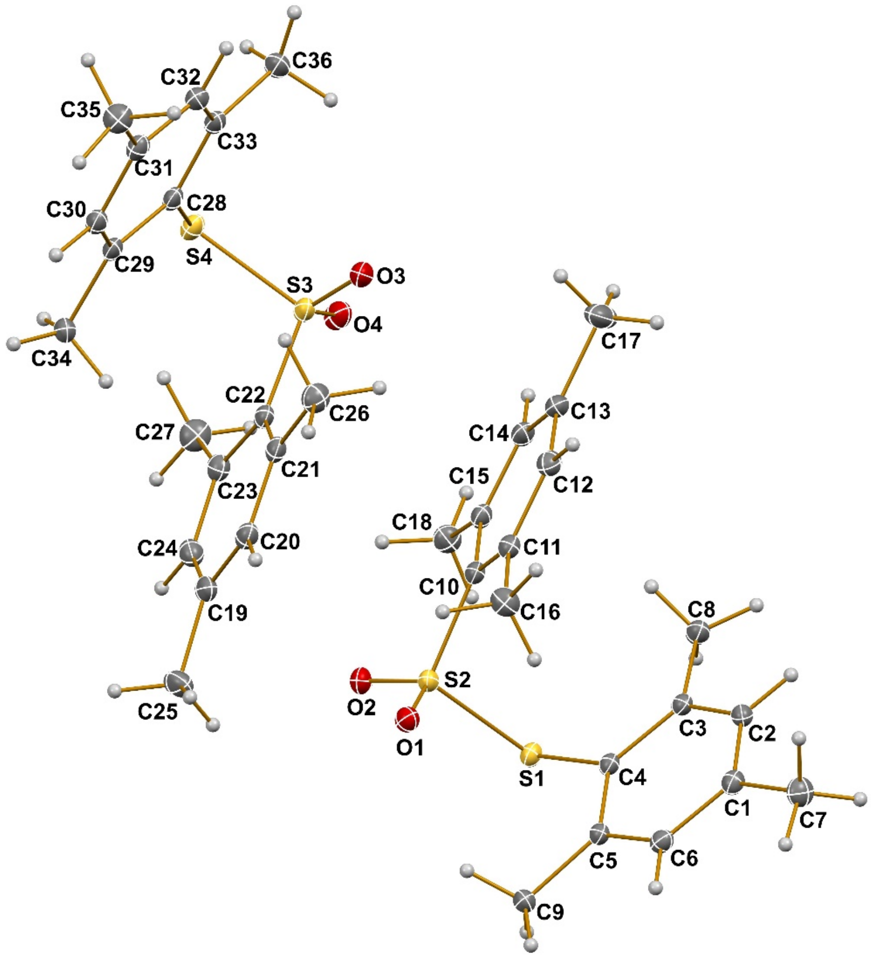

Figure S2. The molecular structure of di-mesityl thiosulfonate at 100(2) K. Atoms are represented by thermal ellipsoids at the 40% probability level. H atoms are shown as spheres of arbitrary radius.

Table S1. Crystallographic data of di-tolyl thiosulfonate and di-mesityl thiosulfonate.

Compound	di-Tolyl thiosulfonate	di-Mesityl thiosulfonate
Empirical formula	$C_{14}H_{14}O_2S_2$	$C_{18}H_{22}O_2S_2$
Formula weight	278.37	334.47
Temperature (K)	100(2)	100(2)
Wavelength (Å)	1.54178	1.54178
Crystal system	Monoclinic	Triclinic
Space group	$P2_1/c$	*P*-1
a (Å)	13.1027(2)	8.55217(11)
b (Å)	6.48350(10)	12.07198(12)
c (Å)	15.1844(3)	17.47625(15)
α (°)	90.00	108.5648(9)
β (°)	92.268(2)	90.3715(10)
γ (°)	90.00	96.8482(9)
V (Å^3)	1288.92(4)	1696.29(3)
Z	4	4
ρ_{calcd} (g·cm^{-3})	1.435	1.310
μ (mm^{-1})	3.667	2.872
F(000)	584	712
Crystal size (mm)	0.03×0.11×0.16	0.03×0.03×0.05
θ range for data collection (°)	3.376-79.346	2.670-80.211
Reflections collected	19630	54241
Independent reflections	2760 [R_{int} = 0.0234]	7328 [R_{int} = 0.0363]
Transmission factors (min/max)	0.66024/1.00000	0.80634/1.00000
Data/restraints/params.	2760/0/164	7328/0/428
$R1$,[a] $wR2$[b] ($I > 2\sigma(I)$)	0.0306, 0.0812	0.0366, 0.0943
$R1$,[a] $wR2$[b] (all data)	0.0310, 0.0815	0.0393 0.0959
Quality-of-fit[c]	1.082	1.072

$R_{int} = \Sigma|F_o^2 - \langle F_o^2 \rangle|/\Sigma|F_o^2|$

[a] $R1 = \Sigma||F_o|-|F_c||/\Sigma|F_o|$. [b] $wR2 = [\Sigma[w(F_o^2-F_c^2)^2]/\Sigma[w(F_o^2)^2]]$.

[c] Quality-of-fit = $[\Sigma[w(F_o^2-F_c^2)^2]/(N_{obs}-N_{params})]^{½}$, based on all data.

Table S2. Bond distances and angles (Å, º) for di-tolyl thiosulfonate

S1—S2	2.0985 (5)	C4—C5	1.3935 (19)
S1—O1	1.4329 (10)	C5—C6	1.3962 (18)
S1—O2	1.4423 (10)	C13—C14	1.3881 (19)
S1—C5	1.7640 (13)		
S2—C12	1.7768 (13)		
C1—C2	1.508 (2)		
C2—C3	1.397 (2)		
C2—C7	1.398 (2)		
C3—C4	1.387 (2)		
C6—C7	1.387 (2)		
C8—C9	1.5056 (19)		
C9—C10	1.398 (2)		
C9—C14	1.397 (2)		
C10—C11	1.3864 (19)		
C11—C12	1.3955 (18)		
C12—C13	1.3935 (19)		
O1—S1—S2	109.58 (4)	C6—C7—C2	121.33 (13)
O1—S1—O2	119.69 (6)	C10—C9—C8	121.22 (13)
O1—S1—C5	108.65 (6)	C14—C9—C8	120.65 (13)
O2—S1—S2	102.91 (5)	C14—C9—C10	118.13 (12)
O2—S1—C5	109.28 (6)	C11—C10—C9	121.15 (12)
C5—S1—S2	105.82 (4)	C10—C11—C12	119.70 (13)
C12—S2—S1	100.04 (4)	C11—C12—S2	119.62 (10)
C3—C2—C1	120.64 (13)	C13—C12—S2	120.23 (10)
C3—C2—C7	118.63 (13)	C13—C12—C11	120.07 (12)
C7—C2—C1	120.69 (13)	C14—C13—C12	119.39 (12)
C4—C3—C2	121.15 (13)	C13—C14—C9	121.46 (13)
C3—C4—C5	118.78 (12)		
C4—C5—S1	118.90 (10)		
C4—C5—C6	121.42 (12)		
C6—C5—S1	119.63 (10)		

C7—C6—C5	118.56 (13)		
S1—S2—C12—C11	-108.29 (10)	C3—C4—C5—S1	-176.79 (10)
S1—S2—C12—C13	68.32 (11)	C3—C4—C5—C6	0.55 (19)
S1—C5—C6—C7	174.86 (10)	C4—C5—C6—C7	-2.47 (19)
S2—S1—C5—C4	104.18 (10)	C5—C6—C7—C2	1.2 (2)
S2—S1—C5—C6	-73.22 (11)	C7—C2—C3—C4	-3.9 (2)
S2—C12—C13—C14	-173.42 (10)	C8—C9—C10—C11	-178.02 (13)
O1—S1—C5—C4	-138.23 (10)	C8—C9—C14—C13	178.53 (13)
O1—S1—C5—C6	44.38 (12)	C9—C10—C11—C12	-0.02 (19)
O2—S1—C5—C4	-6.01 (12)	C10—C9—C14—C13	-1.6 (2)
O2—S1—C5—C6	176.60 (10)	C10—C11—C12—S2	173.94 (10)
C1—C2—C3—C4	173.91 (13)	C10—C11—C12—C13	-2.68 (19)
C1—C2—C7—C6	-175.90 (13)	C11—C12—C13—C14	3.18 (19)
C2—C3—C4—C5	2.7 (2)	C12—C13—C14—C9	-1.0 (2)
C3—C2—C7—C6	1.93 (19)	C14—C9—C10—C11	2.15 (19)

Table S3. Bond distances and angles (Å, º) for di-mesityl thiosulfonate

S1—S2	2.0973 (5)	C14—C13	1.384 (3)
S1—C4	1.7770 (15)	C23—C24	1.383 (2)
S2—O1	1.4283 (13)	C23—C27	1.511 (2)
S2—O2	1.4359 (13)	C12—C13	1.391 (3)
S2—C10	1.7794 (16)	C20—C19	1.385 (3)
S4—S3	2.0926 (5)	C19—C24	1.390 (3)
S4—C28	1.7719 (17)	C19—C25	1.505 (2)
S3—O4	1.4361 (13)	C13—C17	1.507 (2)
S3—O3	1.4352 (14)	C33—C32	1.388 (2)
S3—C22	1.7787 (16)	C33—C36	1.508 (2)
C5—C4	1.408 (2)	C30—C31	1.388 (2)
C5—C6	1.393 (2)	C22—C21	1.414 (2)
C5—C9	1.507 (2)	C22—C23	1.415 (2)
C3—C4	1.411 (2)	C21—C20	1.394 (2)
C3—C2	1.389 (2)	C21—C26	1.505 (2)
C3—C8	1.508 (2)	C31—C32	1.397 (2)
C10—C11	1.412 (2)	C31—C35	1.507 (2)
C10—C15	1.413 (2)		
C29—C34	1.507 (2)		
C29—C30	1.394 (2)		
C29—C28	1.408 (2)		
C11—C12	1.392 (2)		
C11—C16	1.516 (2)		
C1—C6	1.392 (2)		
C1—C2	1.392 (2)		
C1—C7	1.504 (2)		
C15—C14	1.391 (2)		
C15—C18	1.507 (2)		
C33—C28	1.410 (2)		
C4—S1—S2	101.68 (5)	C19—C20—C21	122.83 (16)
O1—S2—S1	109.66 (6)	C20—C19—C24	118.14 (16)

O1—S2—O2	118.46 (8)	C20—C19—C25	121.72 (17)
O1—S2—C10	109.89 (8)	C24—C19—C25	120.14 (17)
O2—S2—S1	102.04 (6)	C14—C13—C12	118.23 (15)
O2—S2—C10	111.24 (8)	C14—C13—C17	121.13 (17)
C10—S2—S1	104.36 (5)	C12—C13—C17	120.63 (17)
C28—S4—S3	100.97 (5)	C33—C32—C31	121.91 (16)
O4—S3—S4	103.54 (6)	C23—C24—C19	122.49 (16)
O4—S3—C22	108.35 (8)	C15—C10—S2	118.05 (12)
O3—S3—S4	109.06 (6)	C30—C29—C34	118.97 (14)
O3—S3—O4	118.35 (8)	C30—C29—C28	118.02 (15)
O3—S3—C22	110.29 (8)	C28—C29—C34	123.01 (15)
C22—S3—S4	106.46 (5)	C10—C11—C16	125.57 (15)
C4—C5—C9	122.72 (14)	C12—C11—C10	117.37 (15)
C6—C5—C4	117.97 (14)	C12—C11—C16	117.07 (15)
C6—C5—C9	119.30 (14)	C6—C1—C2	118.57 (14)
C4—C3—C8	122.91 (14)	C6—C1—C7	120.83 (15)
C2—C3—C4	117.74 (14)	C2—C1—C7	120.60 (15)
C2—C3—C8	119.33 (14)	C5—C4—S1	118.88 (11)
C11—C10—S2	120.47 (12)	C5—C4—C3	121.51 (14)
C11—C10—C15	121.38 (14)	C3—C4—S1	119.56 (12)
C1—C6—C5	121.92 (14)	C28—C33—C36	122.10 (15)
C10—C15—C18	124.80 (15)	C32—C33—C28	118.27 (14)
C14—C15—C10	117.87 (15)	C32—C33—C36	119.62 (15)
C14—C15—C18	117.33 (14)	C31—C30—C29	122.14 (15)
C21—C22—S3	121.05 (12)	C23—C22—S3	117.67 (12)
C21—C22—C23	121.27 (15)	C3—C2—C1	122.27 (14)
C22—C21—C26	125.59 (15)	C13—C14—C15	122.42 (15)
C20—C21—C22	117.19 (15)	C22—C23—C27	125.06 (16)
C20—C21—C26	117.21 (15)	C24—C23—C22	117.90 (15)
C30—C31—C32	118.48 (15)	C24—C23—C27	117.04 (16)
C30—C31—C35	121.04 (15)	C13—C12—C11	122.74 (16)
C32—C31—C35	120.46 (16)		
C29—C28—S4	120.24 (13)		
C29—C28—C33	121.17 (15)		

C33—C28—S4	118.50 (12)		
S1—S2—C10—C11	-99.63 (12)	C34—C29—C28—S4	2.5 (2)
S1—S2—C10—C15	76.80 (12)	C34—C29—C28—C33	178.98 (14)
S2—S1—C4—C5	85.12 (12)	C30—C29—C28—S4	-177.26 (11)
S2—S1—C4—C3	-97.50 (12)	C30—C29—C28—C33	-0.8 (2)
S2—C10—C11—C12	176.06 (12)	C30—C31—C32—C33	0.5 (2)
S2—C10—C11—C16	-4.1 (2)	C22—C21—C20—C19	-1.1 (2)
S2—C10—C15—C14	-175.92 (12)	C22—C23—C24—C19	1.0 (2)
S2—C10—C15—C18	3.3 (2)	C2—C3—C4—S1	-179.31 (11)
S4—S3—C22—C21	-107.21 (12)	C2—C3—C4—C5	-2.0 (2)
S4—S3—C22—C23	72.07 (12)	C2—C1—C6—C5	-0.7 (2)
S3—S4—C28—C29	-93.59 (12)	C21—C22—C23—C24	-4.4 (2)
S3—S4—C28—C33	89.82 (12)	C21—C22—C23—C27	174.40 (16)
S3—C22—C21—C20	-176.34 (12)	C21—C20—C19—C24	-2.1 (2)
S3—C22—C21—C26	5.3 (2)	C21—C20—C19—C25	177.57 (16)
S3—C22—C23—C24	176.33 (12)	C28—C29—C30—C31	-0.1 (2)
S3—C22—C23—C27	-4.9 (2)	C28—C33—C32—C31	-1.3 (2)
O4—S3—C22—C21	141.99 (13)	C23—C22—C21—C20	4.4 (2)
O4—S3—C22—C23	-38.74 (14)	C23—C22—C21—C26	-173.96 (15)
O3—S3—C22—C21	10.98 (15)	C9—C5—C4—S1	0.0 (2)
O3—S3—C22—C23	-169.74 (12)	C9—C5—C4—C3	-177.30 (14)
O1—S2—C10—C11	17.88 (15)	C9—C5—C6—C1	178.70 (15)
O1—S2—C10—C15	-165.68 (12)	C20—C19—C24—C23	2.1 (3)
O2—S2—C10—C11	151.07 (13)	C8—C3—C4—S1	-0.7 (2)
O2—S2—C10—C15	-32.50 (14)	C8—C3—C4—C5	176.58 (14)
C10—C11—C12—C13	-0.2 (2)	C8—C3—C2—C1	-177.74 (15)
C10—C15—C14—C13	-0.3 (2)	C32—C33—C28—S4	178.04 (11)
C29—C30—C31—C32	0.3 (2)	C32—C33—C28—C29	1.5 (2)
C29—C30—C31—C35	178.74 (15)	C36—C33—C28—S4	-1.1 (2)
C11—C10—C15—C14	0.5 (2)	C36—C33—C28—C29	-177.66 (14)
C11—C10—C15—C18	179.75 (15)	C36—C33—C32—C31	177.83 (15)
C11—C12—C13—C14	0.4 (3)	C18—C15—C14—C13	-179.61 (16)
C11—C12—C13—C17	179.37 (17)	C26—C21—C20—C19	177.41 (15)

C4—C5—C6—C1	-0.4 (2)	C16—C11—C12—C13	179.94 (16)
C4—C3—C2—C1	0.9 (2)	C7—C1—C6—C5	-179.63 (15)
C6—C5—C4—S1	179.08 (11)	C7—C1—C2—C3	179.37 (15)
C6—C5—C4—C3	1.8 (2)	C35—C31—C32—C33	-177.99 (15)
C6—C1—C2—C3	0.4 (2)	C27—C23—C24—C19	-177.86 (16)
C15—C10—C11—C12	-0.3 (2)	C25—C19—C24—C23	-177.53 (16)
C15—C10—C11—C16	179.60 (15)	C34—C29—C30—C31	-179.89 (14)
C15—C14—C13—C12	-0.1 (3)		
C15—C14—C13—C17	-179.12 (17)		

X-ray absorption spectroscopy. Solution samples for XAS were dissolved in toluene at total sulfur concentrations of 100 mM to minimize X-ray fluorescence self-absorption effects;[14] data reported in this paper are essentially free of any self-absorption distortion. Solutions were placed in SPEX CertiPrep (Metuchen NJ, USA) X-cell sample cups employing a 3 µm thick Etnom® window (Chemplex Industries, Inc, Palm City FL, USA) to transmit the X-ray fluorescence. Sulfur K-edge XAS was measured at the Stanford Synchrotron Radiation Lightsource (SSRL) on beamline 4-3 at room temperature, with the sample enclosed in an atmosphere of helium gas to minimize atmospheric attenuation of the X-rays. SSRL beamline 4-3 is equipped with a Si(111) double crystal monochromator and an upstream vertically collimating mirror. The upstream mirror was used to reject harmonic from the monochromatic X-ray beam by setting its angle to give a cutoff energy of approximately 6 keV. Incident X-ray intensities were monitored using a helium-filled gas ionization detector upstream of the sample, with the sweeping voltage adjusted until the output had plateaued. Spectra of solutions were measured by monitoring the total X-ray fluorescence using a large area passivated implanted planar silicon (PIPS) photodiode detector (Canberra Industries, Meriden CT, USA). The PIPS detector is susceptible to background signals from visible light, and these were excluded using an aluminized mylar window on the face of the detector, and by darkening the beamline 4-3 experimental hutch. The incident X-ray energy was calibrated with reference to the spectrum of a solid anhydrous sodium thiosulfate ($Na_2S_2O_3$) standard using the literature value of 2469.2 eV as the energy of the lowest energy K-edge absorption peak.[15]

Density functional theory (DFT) geometry optimizations. DFT geometry optimizations were carried out using DMol^3 and Biovia Materials Studio Version 2022.[16,17] The hybrid PBE0 functional[18] was used both for the potential during the self-consistent field procedure and for the energies. DMol^3 double numerical basis sets included polarization functions for all atoms with all-

---

[14] George, G. N.; Gnida, M.; Bazylinski, D. A.; Prince R. C.; Pickering, I. J. X-ray absorption Spectroscopy as a Probe of Microbial Sulfur Biochemistry: The Nature of Bacterial Sulfur Globules Revisited. *J. Bacteriol.* **2008**, *190*, 6376–6383.

[15] Sekiyama, H.; Kosugi, N.; Kuroda, H.; Ohta, T. Sulfur K-edge Absorption Spectra of Na_2SO_4, Na_2SO_3, $Na_2S_2O_3$, and $Na_2S_2O_x$ (x=5-8). *Bull Chem. Soc. Jpn.* **1986**, *59*, 575–579.

[16] Delley, B. An All-Electron Numerical Method for Solving the Local Density Functional for Polyatomic Molecules. *J. Chem. Phys.* **1990**, *92*, 508–517.

[17] Delley, B. From molecules to solids with the DMol^3 approach. *J. Chem. Phys.* **2000**, *113*, 7756–7764.

[18] Perdew, J. P.; Ernzerhof, E.; Burke, K. Rationale for mixing exact exchange with density functional approximations. *J. Chem. Phys.* **1996**, *105*, 9982–9985.

electron core treatments. DFT geometry optimizations typically show small but systematic variations in bond-lengths from observed values, in most cases the computed values are slightly longer, but when relativistic corrections are applied (where relevant) computed values can be slightly shorter. The choice of functional for our calculations was determined by comparing all GGA, meta-GGA and hybrid functionals available for DMol^3 for S–S, S–O and S–C bond-lengths in compounds from the Cambridge Structure Database (CSD).[19] This evaluation excluded disordered structures, structures with errors, and structures with R>0.05. Hybrid PBE0 gave the best match for crystallographically determined S–S and S–C bond-lengths with maximum respective errors of +0.017 and +0.012 Å, and came second best (after the hybrid-SCAN0 functional) for S–O bond-lengths with a maximum error of +0.026 Å. This functional combines the Perdew–Burke–Ernzerhof (PBE)[20] exchange energy and the Hartree–Fock[21] exchange energy in a three to one ratio, along with the full PBE correlation energy. In DMol^3 solvation effects were included using the Conductor-like Screening Model (COSMO)[22] employing the dielectric constant ε for toluene, was assumed to be 2.38.

Density functional theory simulations of sulfur near-edge spectra. Simulations of near-edge spectra used coordinates obtained from Dmol^3 geometry optimizations and were calculated using the StoBe-deMon code.[23] Calculations employed the non-local exchange functional of Perdew and Wang[24] and the Perdew correlation functional approximation.[25] The (7111/411/1), (6311/311/1) and (311/1) basis sets were used for oxygen, carbon, and hydrogen, respectively. For the excited sulfur atom, we used the relatively large IGLO-III basis set[26] employing the half-core-hole

---

[19] Groom, C. R.; Bruno, I. J.; Lightfoot M. P.; Ward, S. C. The Cambridge Structural Database. *Acta Cryst.* **2016**, *B72*, 171–179.

[20] Perdew, J. P.; Burke, K.; Ernzerhof, M. Generalized Gradient Approximation Made Simple. *Phys. Rev. Lett.* **1996**, *77*, 3865–3868. *Erratum*, **1997**, *78*, 1369.

[21] Becke, A. D. A new mixing of Hartree-Fock and local density-functional theories. *J. Chem. Phys.* **1993**, *98*, 1372–1377.

[22] Klamt, A.; Schüürmann, G. COSMO: A New Approach to Dielectric Screening in Solvents with Explicit Expressions for the Screening Energy and its Gradient. *J. Chem. Soc., Perkin Trans.* **1993**, *2*, 799–805.

[23] Hermann, K.; Pettersson, L. G. M.; Casida, M. E.; Daul, C.; Goursot, A.; Koester, A.; Proynov, E.; St-Amant, A.; Salahub, D. R.; Carravetta, V.; Duarte, H.; Godbout, N.; Guan, J.; Jamorski, C.; Leboeuf, M.; Malkin, V.; Malkina, O.; Nyberg, M.; Pedocchi, L.; Sim, F.; Triguero, L.; Vela, A. StoBe-deMon Code; 2001.

[24] Perdew, J. P.; Wang, Y. Accurate and Simple Density Functional for the Electronic Exchange Energy: Generalized Gradient Approximation. *Phys Rev. B* **1986**, *33*, 8800(R)

[25] Perdew, J. P. Density-Functional Approximation for the Correlation Energy of the Inhomogeneous Electron Gas. *Phys. Rev. B* **1986**, *33*, 8822–8824. Erratum: *Phys. Rev. B* **1986**, *34*, 7406.

[26] Kutzelnigg, W.; Fleischer, U.; Schindler, M. *NMR Basic Principles and Progress*; Springer: Berlin, Heidelberg, New York, **1990**.

approximation, in which half an electron is removed from the 1s level, to generate the potential for computation of the excited states.[27] Calculations were targeted to a particular sulfur atom by using an effective core potential combined with the (311/211/1) basis set for sulfur atom not being excited, which lacks the 1s level. Calculations were done by setting one sulfur atom as the excited atom and then the other. In cases where there is more than one conformation, such as syn and anti-disulfoxide (e.g. 9a), we computed spectra for both sulfur atoms for all possible conformers, with the final simulation representing the sum of these. Interpolation of the exchange-correlation potential employed the auxiliary basis sets (5,4;5,4) for sulfur, (5,2;5,2) for carbon and oxygen, and (3,1;3,1) for hydrogen. To provide alignment with experimental data, calculated transition energies were shifted by +5.35 eV. StoBe-deMon calculated transition intensities were convoluted with a pseudo-Vogt peak-shape function using the ramping-width convolution method, together with an integrated pseudo-Vogt step function (an erf function plus an arctangent) to simulate the edge step. In all simulated spectra reported here the pseudo-Vogt mixing parameter was set to 0.7, and the half-width at half-height varied from 0.6 eV at 2477 eV and below to 50 eV at 2700 eV and above.

[27] Triguero, L.; Pettersson, L. G. M.; Ågren, H. Calculations of near-edge X-ray-absorption spectra of gas-phase and chemisorbed molecules by means of density-functional and transition-potential theory. *Phys. Rev. B.*, **1998**, *58*, 8097–8110.

Table S4. Geometry optimized cartesian coordinates and total energies for the compounds listed in Table 1, plus free radicals required for calculation of BDE values.

Convergence criteria:
Energy = 1.0E-05 Ha
Maximum Force = 0.02 Ha/Å
Maximum Displacement = 0.005 Å
Maximum iterations = 150
Maximum step size = 0.3 Å
SCF tolerance = 1.0E-06
Maximum SCF cycles = 250
No imaginary frequencies

MeS(O)S(O)Me *syn*

ATOM		X	Y	Z
1	S	-2.458890	0.428916	1.250542
2	S	-1.363020	0.722483	-0.716942
3	O	-2.182739	0.186295	-1.859194
4	O	-3.320287	1.646224	1.400211
5	C	-0.111612	-0.521411	-0.332681
6	C	-3.490780	-0.907892	0.615634
7	H	0.572235	-0.536946	-1.178027
8	H	-0.570781	-1.500727	-0.207426
9	H	0.412064	-0.211033	0.571901
10	H	-2.926876	-1.836026	0.713285
11	H	-3.750984	-0.720991	-0.427633
12	H	-4.369919	-0.935401	1.255071

Total Energy = -1020.6838589 Ha

MeS(O)S(O)Me *anti*

ATOM		X	Y	Z
1	S	-2.551629	0.367438	1.442857
2	S	-1.356408	0.762248	-0.436946

3	O	-0.691353	2.080575	-0.187471
4	O	-3.518950	1.509749	1.494665
5	C	-0.124750	-0.523216	-0.144406
6	C	-3.435703	-1.046501	0.734013
7	H	0.616812	-0.413840	-0.934460
8	H	-0.598382	-1.501431	-0.220620
9	H	0.337651	-0.382563	0.829745
10	H	-2.769582	-1.907999	0.709693
11	H	-3.805323	-0.785347	-0.255672
12	H	-4.272044	-1.241126	1.403693

Total Energy = -1020.6802814 Ha

MeS(O)SMe

	ATOM	X	Y	Z
1	S	-2.502353	0.521175	1.074580
2	S	-1.358496	0.642548	-0.730032
3	O	-3.465125	1.669522	1.067156
4	C	-0.087106	-0.572687	-0.285827
5	C	-3.486747	-0.931644	0.650504
6	H	0.621296	-0.570882	-1.113207
7	H	-0.505847	-1.572950	-0.193859
8	H	0.425029	-0.286455	0.631699
9	H	-2.825889	-1.779837	0.477569
10	H	-4.079966	-0.696209	-0.229791
11	H	-4.125238	-1.128616	1.512673

Total Energy = -946.0975438 Ha

MeSO$_2$SMe

	ATOM	X	Y	Z
1	C	-3.713285	-0.192205	-0.127249
2	S	-2.602212	-1.320200	0.673907
3	S	-0.947378	-0.059269	0.949679
4	C	0.096165	-1.296754	1.780947

5	H	-4.619830	-0.760956	-0.327767
6	H	-3.254352	0.137435	-1.057637
7	H	0.289170	-2.135100	1.115360
8	H	-0.371846	-1.623210	2.707724
9	H	1.029276	-0.781565	2.003624
10	H	-3.917112	0.634945	0.548702
11	O	-2.244380	-2.373208	-0.266190
12	O	-3.142633	-1.696881	1.971467

Total Energy = -1020.7201911 Ha

MeSSMe

ATOM		X	Y	Z
1	C	-4.603555	0.656405	1.617477
2	S	-4.248856	0.336110	-0.129435
3	S	-2.514517	-0.753499	-0.065434
4	C	-1.232439	0.517707	0.106851
5	H	-4.716909	-0.267908	2.178030
6	H	-5.539493	1.215096	1.636574
7	H	-3.819948	1.272103	2.051054
8	H	-1.221419	1.190259	-0.747493
9	H	-0.285906	-0.020242	0.176158
10	H	-1.392954	1.064676	1.031383

Total Energy = -871.5166318 Ha

MeS·

ATOM		X	Y	Z
1	C	-3.717550	-0.229315	-0.144556
2	S	-2.575445	-1.322299	0.707786
3	H	-4.666920	-0.719630	-0.348001
4	H	-3.271883	0.169491	-1.055025
5	H	-3.894052	0.609567	0.534944

Total Energy = -435.7079741 Ha

MeS(O)·

ATOM		X	Y	Z
1	S	-2.644483	0.146925	1.729974
2	O	-3.350111	1.464692	1.455332
3	C	-3.486993	-1.084747	0.731576
4	H	-3.044760	-2.055001	0.963251
5	H	-3.346320	-0.856742	-0.324795
6	H	-4.546333	-1.083363	0.981980

Total Energy = -510.3269253 Ha

$MeSO_2$·

ATOM		X	Y	Z
1	C	-3.729657	-0.195849	-0.131847
2	S	-2.601515	-1.337780	0.683542
3	H	-4.640940	-0.752739	-0.340027
4	H	-3.238342	0.121149	-1.048081
5	H	-3.906937	0.631905	0.548007
6	O	-2.244545	-2.376085	-0.298613
7	O	-3.160043	-1.692681	1.998206

Total Energy = -584.9252631 Ha

PhS(O)S(O)Ph *syn*

ATOM		X	Y	Z
**1**	**S**	**-2.530871**	**0.432385**	**1.294550**
2	S	-1.270673	0.873163	-0.592838
3	O	-2.111227	0.610643	-1.803205
4	O	-3.466467	1.601460	1.344386
5	C	-0.143448	-0.489497	-0.375287
6	C	-3.404299	-0.948470	0.598264
7	C	-4.584788	-0.747965	-0.105613
8	C	-5.264694	-1.854991	-0.589178

9	C	-4.771685	-3.133854	-0.367623
10	C	-3.587783	-3.320199	0.334856
11	C	-2.900806	-2.223181	0.829886
12	C	-0.179702	-1.558671	-1.257340
13	C	0.751520	-2.576279	-1.109066
14	C	1.696837	-2.517468	-0.093810
15	C	1.723547	-1.436193	0.780738
16	C	0.804036	-0.409495	0.638871
17	H	-4.961455	0.255750	-0.251499
18	H	-6.186711	-1.714700	-1.139355
19	H	-3.203711	-4.317542	0.507147
20	H	-1.991295	-2.360330	1.400975
21	H	-0.921998	-1.578686	-2.044542
22	H	0.736091	-3.416905	-1.790813
23	H	2.463917	-1.385687	1.568342
24	H	0.830814	0.446438	1.303183
25	H	-5.314290	-3.989042	-0.749569
26	H	2.418973	-3.315980	0.012011

Total Energy = -1400.3552399 Ha

PhS(O)S(O)Ph *anti*

ATOM		X	Y	Z
1	S	-2.480180	0.169917	1.480515
2	S	-1.327043	0.826377	-0.425271
3	O	-0.730261	2.141954	-0.037350
4	O	-3.376536	1.341812	1.727067
5	C	-0.091934	-0.440546	-0.228027
6	C	-3.430274	-1.109063	0.652079
7	C	-4.554686	-0.784139	-0.101721
8	C	-5.281238	-1.802413	-0.699502
9	C	-4.897628	-3.128873	-0.518207
10	C	-3.788173	-3.442609	0.256005
11	C	-3.056590	-2.432141	0.864400
12	C	-0.355567	-1.695376	-0.758830
13	C	0.601034	-2.688652	-0.627085

14	C	1.802851	-2.416783	0.015380
15	C	2.054165	-1.149636	0.527848
16	C	1.099099	-0.152351	0.422483
17	H	-4.841885	0.253779	-0.217990
18	H	-6.149899	-1.562799	-1.301067
19	H	-3.503778	-4.477317	0.402232
20	H	-2.199261	-2.667411	1.483000
21	H	-1.274925	-1.880536	-1.299941
22	H	0.417874	-3.666639	-1.052168
23	H	2.992552	-0.940784	1.024827
24	H	1.265795	0.841166	0.820899
25	H	-5.462324	-3.921589	-0.995388
26	H	2.554635	-3.191947	0.099727

Total Energy = -1400.3519958 Ha

PhS(O)SO_2Ph

	ATOM	X	Y	Z
1	S	-2.743222	0.625394	1.339654
2	S	-1.190433	0.960615	-0.218954
3	O	-1.904239	1.035612	-1.484125
4	O	-0.457435	2.093946	0.320684
5	C	-0.159212	-0.456039	-0.187629
6	C	-3.371906	-0.897531	0.660459
7	C	-4.316701	-0.826692	-0.355941
8	C	-4.808903	-2.004281	-0.893681
9	C	-4.363357	-3.228387	-0.410844
10	C	-3.433206	-3.280991	0.619990
11	C	-2.933495	-2.110411	1.167348
12	C	-0.332331	-1.443960	-1.149401
13	C	0.519364	-2.535933	-1.135272
14	C	1.504596	-2.636372	-0.159794
15	C	1.651317	-1.647510	0.806265
16	C	0.815335	-0.542416	0.800105
17	H	-4.657862	0.133472	-0.722053
18	H	-5.542210	-1.967223	-1.688953

19	H	-3.098078	-4.237557	0.999971
20	H	-2.214883	-2.122918	1.977000
21	H	-1.102262	-1.336171	-1.901483
22	H	0.416623	-3.306875	-1.887554
23	H	2.421623	-1.731919	1.561452
24	H	0.925279	0.249808	1.527393
25	O	-2.009564	0.310197	2.604715
26	H	-4.749564	-4.145605	-0.836906
27	H	2.170757	-3.489606	-0.156361

Total Energy = -1474.9707119 Ha

PhS(O)SPh

ATOM		X	Y	Z
1	S	-2.591980	0.194291	1.473725
2	S	-1.290006	0.807020	-0.161045
3	O	-3.504145	1.373427	1.624478
4	C	-0.038269	-0.447316	-0.118920
5	C	-3.555021	-1.102640	0.676661
6	C	-4.589642	-0.799425	-0.202164
7	C	-5.294896	-1.834417	-0.792482
8	C	-4.964642	-3.156474	-0.499305
9	C	-3.947519	-3.444508	0.399534
10	C	-3.236107	-2.413792	1.001338
11	C	-0.276082	-1.680463	-0.720414
12	C	0.711883	-2.652114	-0.717635
13	C	1.946187	-2.389752	-0.139351
14	C	2.189408	-1.154263	0.446632
15	C	1.199142	-0.183639	0.464979
16	H	-4.827406	0.236238	-0.409894
17	H	-6.095420	-1.611990	-1.487472
18	H	-3.703996	-4.474267	0.628275
19	H	-2.446177	-2.629982	1.710462
20	H	-1.225632	-1.859012	-1.207478
21	H	0.527276	-3.604351	-1.198108
22	H	3.150386	-0.949375	0.902304
23	H	1.375581	0.774436	0.936731

24	H	-5.510571	-3.962665	-0.974990
25	H	2.720279	-3.146661	-0.149756

Total Energy = -1325.7686540 Ha

PhSO₂SO₂Ph

ATOM		X	Y	Z
1	S	-2.616983	0.363366	1.329739
2	S	-1.161624	0.887464	-0.240488
3	O	-1.896041	0.806114	-1.488483
4	O	-0.568353	2.120007	0.235198
5	C	-0.007759	-0.425498	-0.147343
6	C	-3.473651	-0.997158	0.614420
7	C	-4.482398	-0.748586	-0.310250
8	C	-5.120278	-1.822528	-0.906730
9	C	-4.743858	-3.120425	-0.576466
10	C	-3.736724	-3.350530	0.351778
11	C	-3.084296	-2.287137	0.953692
12	C	-0.310799	-1.628220	-0.776945
13	C	0.595615	-2.670299	-0.674113
14	C	1.768151	-2.510021	0.056877
15	C	2.056009	-1.298084	0.673548
16	C	1.150909	-0.252372	0.597906
17	H	-4.756866	0.269785	-0.547132
18	H	-5.911834	-1.642983	-1.622856
19	H	-3.462034	-4.365607	0.607586
20	H	-2.296309	-2.450743	1.675714
21	H	-1.214395	-1.728591	-1.362952
22	H	0.394141	-3.607154	-1.177392
23	H	2.971458	-1.170925	1.235722
24	H	1.331740	0.689899	1.096491
25	O	-1.848830	-0.092743	2.471743
26	O	-3.494880	1.514951	1.381881
27	H	-5.245044	-3.957247	-1.045961
28	H	2.471161	-3.329467	0.126738

Total Energy = -1549.5806738 Ha

$PhSO_2SPh$

	ATOM	X	Y	Z
1	S	-2.722884	0.609827	1.320272
2	S	-1.292400	0.959692	-0.166982
3	O	-1.945491	0.988555	-1.461417
4	O	-0.583758	2.116925	0.350985
5	C	-0.207944	-0.431055	-0.164484
6	C	-3.436102	-0.867863	0.653662
7	C	-4.361408	-0.798914	-0.383460
8	C	-4.887045	-1.969250	-0.904934
9	C	-4.497888	-3.199249	-0.390541
10	C	-3.578787	-3.262089	0.648263
11	C	-3.045781	-2.096450	1.173589
12	C	-0.368636	-1.417760	-1.127209
13	C	0.529579	-2.475293	-1.153714
14	C	1.564539	-2.533705	-0.229064
15	C	1.709286	-1.538044	0.731749
16	C	0.818601	-0.476954	0.769825
17	H	-4.646893	0.164674	-0.784343
18	H	-5.608006	-1.919110	-1.711247
19	H	-3.279326	-4.220361	1.053644
20	H	-2.324679	-2.133222	1.979593
21	H	-1.169483	-1.342386	-1.849526
22	H	0.424832	-3.248289	-1.904662
23	H	2.522832	-1.581881	1.444213
24	H	0.926395	0.316324	1.497830
25	H	-4.913979	-4.110045	-0.801999
26	H	2.267637	-3.356396	-0.259163

Total Energy = -1400.3932771 Ha

PhSSPh

ATOM		X	Y	Z
1	S	-2.512875	0.354996	0.929297
2	S	-1.231440	0.611978	-0.672096
3	C	0.069630	-0.571919	-0.387446
4	C	-3.569554	-0.998650	0.429773
5	C	-4.851234	-0.732351	-0.040859
6	C	-5.694398	-1.787407	-0.364546
7	C	-5.258528	-3.097365	-0.215565
8	C	-3.978637	-3.359478	0.254776
9	C	-3.136294	-2.311075	0.589275
10	C	0.245699	-1.596521	-1.309097
11	C	1.296345	-2.490941	-1.151677
12	C	2.155804	-2.369046	-0.068632
13	C	1.967078	-1.350064	0.858477
14	C	0.930827	-0.444878	0.698318
15	H	-5.184360	0.291756	-0.152494
16	H	-6.696803	-1.586001	-0.724964
17	H	-3.638075	-4.380555	0.370416
18	H	-2.140143	-2.499581	0.967803
19	H	-0.438258	-1.690996	-2.142442
20	H	1.437590	-3.285943	-1.873867
21	H	2.638356	-1.252679	1.702690
22	H	0.783454	0.359343	1.408703
23	H	-5.919779	-3.918705	-0.465168
24	H	2.974055	-3.068248	0.054345

Total Energy = -1251.1905897 Ha

PhS·

ATOM		X	Y	Z
1	S	-2.755813	0.603456	1.304042
2	C	-3.419516	-0.848220	0.663944
3	C	-4.356480	-0.800698	-0.387567
4	C	-4.886803	-1.964372	-0.905420

5	C	-4.499205	-3.197941	-0.388951
6	C	-3.574388	-3.263915	0.651047
7	C	-3.038499	-2.104719	1.174153
8	H	-4.643960	0.165569	-0.780956
9	H	-5.603892	-1.916619	-1.715191
10	H	-3.278474	-4.225991	1.050203
11	H	-2.319929	-2.142233	1.982098
12	H	-4.925819	-4.106367	-0.794902

Total Energy = -625.5572413 Ha

PhSO·

ATOM		X	Y	Z
1	S	-2.585404	0.356348	1.460071
2	O	-3.336607	1.646259	1.206872
3	C	-3.464532	-0.958415	0.684611
4	C	-4.646173	-0.735622	-0.018160
5	C	-5.300284	-1.816001	-0.586406
6	C	-4.785682	-3.101771	-0.446953
7	C	-3.601087	-3.312217	0.250945
8	C	-2.935525	-2.242370	0.823959
9	H	-5.027078	0.274273	-0.111483
10	H	-6.218714	-1.655729	-1.137704
11	H	-3.198513	-4.313297	0.344108
12	H	-2.013708	-2.399221	1.372577
13	H	-5.304127	-3.940623	-0.894099

Total Energy = -700.1668183 Ha

PhSO$_2$·

ATOM		X	Y	Z
1	S	-1.310770	0.938870	-0.170327
2	O	-1.964834	1.054178	-1.482645
3	O	-0.666382	2.085488	0.488019
4	C	-0.162421	-0.411333	-0.215698
5	C	-0.348065	-1.417744	-1.152873

6	C	0.530187	-2.490034	-1.148699
7	C	1.557400	-2.548259	-0.213838
8	C	1.718610	-1.534120	0.723047
9	C	0.852107	-0.452865	0.731458
10	H	-1.148603	-1.347147	-1.876302
11	H	0.411637	-3.281266	-1.877252
12	H	2.523023	-1.578648	1.445149
13	H	0.968468	0.354259	1.441652
14	H	2.235631	-3.391645	-0.213311

Total Energy = -774.7599018 Ha

***p*-TolS(O)S(O)Tol-*p*, *syn*-9a**

ATOM		X	Y	Z
1	S	-2.526325	0.400534	1.337870
2	S	-1.301946	0.828573	-0.596831
3	O	-2.171143	0.519525	-1.776725
4	O	-3.447489	1.579506	1.425175
5	C	-0.146409	-0.499999	-0.379921
6	C	-3.423172	-0.947677	0.632683
7	C	-4.606389	-0.721880	-0.059918
8	C	-5.283418	-1.806489	-0.589505
9	C	-4.798910	-3.105946	-0.435073
10	C	-3.610339	-3.301629	0.269509
11	C	-2.920890	-2.230826	0.810746
12	C	-0.190934	-1.611789	-1.208094
13	C	0.759670	-2.607112	-1.042473
14	C	1.753376	-2.501527	-0.069913
15	C	1.776497	-1.366664	0.745300
16	C	0.838649	-0.361968	0.591107
17	C	-5.545867	-4.273361	-1.003245
18	C	2.790217	-3.567144	0.088635
19	H	-4.987063	0.286242	-0.164278
20	H	-6.202452	-1.642012	-1.140239
21	H	-3.219394	-4.304325	0.400986
22	H	-2.010360	-2.387767	1.376201

23	H	-0.953519	-1.683567	-1.973224
24	H	0.726212	-3.484143	-1.678974
25	H	2.543174	-1.268216	1.505444
26	H	0.884630	0.528466	1.207968
27	H	-4.876790	-5.105200	-1.222376
28	H	-6.072269	-3.999728	-1.917358
29	H	-6.292188	-4.629145	-0.289159
30	H	3.624531	-3.406860	-0.597256
31	H	2.375889	-4.553975	-0.122712
32	H	3.193318	-3.579337	1.101137

Total Energy = -1478.1769005 Ha

***p*-TolS(O)S(O)Tol-*p*, *anti*-9a**

ATOM		X	Y	Z
1	S	-2.549123	0.230705	1.523500
2	S	-1.350139	0.825488	-0.356045
3	O	-0.736806	2.135091	0.027811
4	O	-3.459353	1.407046	1.698814
5	C	-0.114399	-0.440294	-0.215992
6	C	-3.457400	-1.068721	0.708990
7	C	-4.521210	-0.779559	-0.140362
8	C	-5.197984	-1.821753	-0.747924
9	C	-4.837495	-3.152044	-0.512344
10	C	-3.776666	-3.409886	0.356828
11	C	-3.087941	-2.380069	0.977711
12	C	-0.360983	-1.666116	-0.822483
13	C	0.598168	-2.658938	-0.738661
14	C	1.797191	-2.440819	-0.060485
15	C	2.027070	-1.192072	0.517917
16	C	1.077531	-0.189106	0.451748
17	C	-5.596431	-4.271765	-1.159071
18	C	2.835122	-3.510593	0.009570
19	H	-4.816387	0.249579	-0.308353
20	H	-6.019807	-1.601170	-1.419447
21	H	-3.486967	-4.434305	0.559260

22	H	-2.276908	-2.592186	1.664667
23	H	-1.273875	-1.827308	-1.383929
24	H	0.417965	-3.613338	-1.220681
25	H	2.955127	-1.016934	1.050367
26	H	1.250884	0.779487	0.905534
27	H	-4.966743	-5.147908	-1.315942
28	H	-6.008683	-3.962971	-2.119695
29	H	-6.433294	-4.578133	-0.525634
30	H	3.498502	-3.460358	-0.857265
31	H	2.382074	-4.502692	0.017377
32	H	3.446442	-3.404882	0.905025

Total Energy = -1478.1742377 Ha

p*-TolS(O)SO_2Tol-*p

ATOM		X	Y	Z
1	S	-2.662531	0.346540	1.449749
2	S	-1.230109	0.833629	-0.213095
3	O	-1.983623	0.755906	-1.456012
4	O	-0.641163	2.083613	0.241415
5	C	-0.030484	-0.433910	-0.150124
6	C	-3.477784	-1.036284	0.674428
7	C	-4.495533	-0.775744	-0.235827
8	C	-5.168824	-1.833106	-0.820637
9	C	-4.853702	-3.154811	-0.496041
10	C	-3.834120	-3.383187	0.430293
11	C	-3.144258	-2.336823	1.023872
12	C	-0.302694	-1.654060	-0.759461
13	C	0.656444	-2.647572	-0.713569
14	C	1.880545	-2.434037	-0.079283
15	C	2.114878	-1.208890	0.543244
16	C	1.161100	-0.209536	0.527814
17	C	-5.637703	-4.285743	-1.097882
18	C	2.942862	-3.480035	-0.100667
19	H	-4.752893	0.244758	-0.488604
20	H	-5.958136	-1.626302	-1.533048

21	H	-3.577964	-4.402749	0.694695
22	H	-2.360103	-2.518995	1.747590
23	H	-1.233660	-1.806202	-1.287109
24	H	0.464698	-3.597254	-1.198872
25	H	3.055265	-1.042432	1.054283
26	H	1.332475	0.737538	1.019411
27	H	-5.044728	-5.197656	-1.177174
28	H	-6.005972	-4.026512	-2.091756
29	H	-6.507776	-4.511224	-0.474680
30	H	3.560978	-3.353584	-0.993322
31	H	2.521181	-4.485654	-0.132866
32	H	3.596487	-3.405699	0.768621
33	O	-1.827665	-0.174183	2.578035

Total Energy = -1552.7903512 Ha

***p*-TolS(O)STol-*p*, 6a**

ATOM		X	Y	Z
1	S	-2.465006	-0.025536	1.542940
2	S	-1.359486	0.639844	-0.216878
3	O	-0.811773	1.990068	0.118972
4	C	-0.002182	-0.514762	-0.135837
5	C	-3.481528	-1.228006	0.728754
6	C	-4.504018	-0.836495	-0.135510
7	C	-5.290813	-1.788970	-0.759947
8	C	-5.093654	-3.150908	-0.523270
9	C	-4.070519	-3.527345	0.346970
10	C	-3.264987	-2.583607	0.966411
11	C	-0.208324	-1.787885	-0.646006
12	C	0.824266	-2.707501	-0.596049
13	C	2.062628	-2.358665	-0.061701
14	C	2.245785	-1.066215	0.425459
15	C	1.218774	-0.138177	0.396087
16	C	-5.978702	-4.172714	-1.172151
17	C	3.183920	-3.343412	-0.037298
18	H	-4.670652	0.217755	-0.318603

19	H	-6.076247	-1.467631	-1.434544
20	H	-3.895632	-4.580473	0.538358
21	H	-2.464242	-2.893220	1.625809
22	H	-1.160408	-2.056781	-1.087116
23	H	0.671341	-3.706369	-0.988672
24	H	3.207067	-0.786450	0.841853
25	H	1.349764	0.865080	0.782596
26	H	-5.455001	-5.117452	-1.319181
27	H	-6.341074	-3.821997	-2.138728
28	H	-6.852792	-4.373165	-0.546124
29	H	3.753621	-3.302564	-0.969932
30	H	2.813167	-4.363306	0.074793
31	H	3.873456	-3.135640	0.781166

Total Energy = -1403.5917112 Ha

***p*-TolSO$_2$STol-*p*, 7a**

ATOM		X	Y	Z
1	S	-2.611391	0.315867	1.257880
2	S	-1.227744	0.775066	-0.253585
3	O	-1.876938	0.677765	-1.547175
4	O	-0.648973	2.021923	0.216097
5	C	0.004681	-0.482341	-0.172581
6	C	-3.496026	-1.039413	0.528483
7	C	-4.603703	-0.791961	-0.278099
8	C	-5.360837	-1.849604	-0.754010
9	C	-5.029869	-3.167974	-0.442754
10	C	-3.896260	-3.397288	0.334671
11	C	-3.132024	-2.348589	0.825370
12	C	-0.064401	-1.565228	-1.039918
13	C	0.933247	-2.525344	-0.991692
14	C	1.994624	-2.416003	-0.093287
15	C	2.023891	-1.328285	0.780580
16	C	1.035340	-0.360646	0.750799
17	C	-5.900763	-4.303241	-0.897192
18	C	3.102503	-3.420366	-0.092490

19	H	-4.871177	0.228205	-0.518084
20	H	-6.222901	-1.648973	-1.377866
21	H	-3.608561	-4.415636	0.564297
22	H	-2.262271	-2.541026	1.440308
23	H	-0.879283	-1.642428	-1.746706
24	H	0.892740	-3.366603	-1.672876
25	H	2.842710	-1.228886	1.482917
26	H	1.069482	0.488416	1.420556
27	H	-5.339294	-5.233381	-0.984680
28	H	-6.365030	-4.085953	-1.859797
29	H	-6.706255	-4.469885	-0.176550
30	H	3.873377	-3.128844	-0.810956
31	H	2.748277	-4.411568	-0.377738
32	H	3.577067	-3.489628	0.886369

Total Energy = -1478.2148162 Ha

***p*-TolSSTol-*p*, 8a**

ATOM		X	Y	Z
1	S	-2.552116	-0.108488	1.515817
2	S	-1.303789	0.629437	0.027646
3	C	0.051049	-0.526685	-0.012380
4	C	-3.585492	-1.300263	0.684962
5	C	-4.491822	-0.911333	-0.297606
6	C	-5.302936	-1.850375	-0.908966
7	C	-5.245152	-3.195840	-0.542342
8	C	-4.336727	-3.569930	0.446538
9	C	-3.508869	-2.638316	1.057389
10	C	-0.137332	-1.824237	-0.479985
11	C	0.927103	-2.708053	-0.518734
12	C	2.201797	-2.310722	-0.118938
13	C	2.381040	-1.004954	0.329008
14	C	1.318002	-0.116262	0.389179
15	C	-6.161944	-4.195408	-1.179622
16	C	3.355084	-3.257453	-0.195671
17	H	-4.549099	0.131066	-0.585387

18	H	-6.002679	-1.531846	-1.673278
19	H	-4.260743	-4.613227	0.733758
20	H	-2.788415	-2.950406	1.803668
21	H	-1.122221	-2.141819	-0.799230
22	H	0.769706	-3.723364	-0.868581
23	H	3.367775	-0.680572	0.643227
24	H	1.466028	0.890908	0.760691
25	H	-5.712618	-5.188236	-1.203078
26	H	-6.419825	-3.906094	-2.197955
27	H	-7.094814	-4.278938	-0.615740
28	H	3.825829	-3.221008	-1.182280
29	H	3.032967	-4.286303	-0.032918
30	H	4.120885	-3.013336	0.540447

Total Energy = -1329.0110004 Ha

***p*-TolS·**

ATOM		X	Y	Z
1	S	-2.714301	0.447364	1.312068
2	C	-3.472894	-0.941877	0.649440
3	C	-4.483656	-0.810916	-0.325664
4	C	-5.116016	-1.921410	-0.839604
5	C	-4.778063	-3.209216	-0.408413
6	C	-3.760604	-3.344898	0.543919
7	C	-3.117138	-2.240767	1.065528
8	C	-5.522860	-4.396106	-0.934633
9	H	-4.749473	0.184352	-0.656941
10	H	-5.890650	-1.796765	-1.587359
11	H	-3.477185	-4.336154	0.878709
12	H	-2.330958	-2.356682	1.801078
13	H	-4.949468	-5.315758	-0.817453
14	H	-5.767685	-4.267809	-1.989783
15	H	-6.465326	-4.523045	-0.394216

Total Energy = -664.4692745 Ha

***p*-TolS(O)·**

ATOM		X	Y	Z
1	S	-2.574144	0.373961	1.458235
2	O	-3.277902	1.673389	1.125488
3	C	-3.457057	-0.945534	0.700865
4	C	-4.647360	-0.735055	0.009892
5	C	-5.304036	-1.816737	-0.548149
6	C	-4.801372	-3.114214	-0.430135
7	C	-3.610451	-3.301696	0.273604
8	C	-2.940514	-2.233602	0.843325
9	C	-5.531558	-4.272810	-1.035252
10	H	-5.040481	0.269581	-0.078431
11	H	-6.226907	-1.652843	-1.091705
12	H	-3.205382	-4.301960	0.375234
13	H	-2.020283	-2.399943	1.391383
14	H	-4.851463	-5.090524	-1.275167
15	H	-6.053377	-3.979312	-1.947019
16	H	-6.281028	-4.662404	-0.340150

Total Energy = -739.0777128 Ha

***p*-TolSO$_2$·**

ATOM		X	Y	Z
1	S	-1.226472	0.930229	-0.214513
2	O	-1.885033	0.982120	-1.529192
3	O	-0.618245	2.118726	0.402940
4	C	-0.045541	-0.382324	-0.217729
5	C	-0.213257	-1.438936	-1.102969
6	C	0.702421	-2.476424	-1.071228
7	C	1.763536	-2.473428	-0.164564
8	C	1.886558	-1.407282	0.727428
9	C	0.984062	-0.359058	0.713418
10	C	2.772168	-3.574831	-0.168448
11	H	-1.031181	-1.435597	-1.810534
12	H	0.589161	-3.303332	-1.761589

13	H	2.702807	-1.398320	1.439031
14	H	1.085852	0.474303	1.394374
15	H	3.579650	-3.347352	-0.869295
16	H	2.325041	-4.519753	-0.479918
17	H	3.218988	-3.710906	0.816402

Total Energy = -813.6710814 Ha

Comparison of ^{1}H, ^{13}C and IR data for tolyl and mesityl disulfides and *S*-oxides

Table S5. Comparison of ^{1}H NMR data (ppm) for Di-*p*-tolyl Thiosulfinate

Souto[a]	7.57 (d, 8.3)	7.44 (d, 8.1)	7.30 (d, 8.0)	7.19 (d, 7.9)	2.42, s	2.38, s
This work[b]	7.59 (d, 8.3)	7.46 (d, 8.0)	7.33 (d, 7.9)	7.22 (d, 7.8)	2.44, s	2.41, s

[a]400 MHz in $CDCl_3$; [b]500 MHz in $CDCl_3$.

Table S6. Comparison of ^{13}C and ^{1}H NMR data for Dimesityl Disulfide[g]

^{13}C Souto[a]	^{13}C Zheng[b]	^{13}C Block[c]	^{1}H Souto[d]	^{1}H Zheng[e]	^{1}H This work[f]
143.2	143.3	143.3	6.84	6.87	6.87
139.2	139.3	139.3	2.25	2.29	2.29
131.6	131.6	131.6	2.21	2.24	2.24
128.9	128.9	128.9			
21.4	21.4	21.5			
21.1	21.2	21.2			

[a]100 MHz; [b]101 MHz; [c]125 MHz; [d]400 MHz; [e]400MHz; [f]500MHz. All spectra run in $CDCl_3$. [g]Names correspond to those in reference list.

Table S7. Comparison of ^{13}C NMR data (ppm) for *p*-$TolSO_2STol$-*p*[a] and Tol "disulfoxide"

Souto[b]	Cai	Nair	Iwata	Cao	Zheng	Ji	Shyam	This work
144.6	144.1	144.4	144.5	144.6	144.6	144.6	144.5	144.6
142.0	141.6	141.9	142.0	142.0	142.1	142.0	142.0	142.1
140.5	140.1	140.9	140.5	140.4	140.4	140.4	140.3	140.5
136.5	136.1	136.5	136.5	136.5	136.5	136.5	136.4	136.5
130.2	129.8	130.1	130.2	130.2	130.2	130.2	130.2	130.2
129.4	128.9	129.3	129.3	129.4	129.4	129.3	129.3	129.4
127.6	127.2	127.7	127.6	127.6	127.6	127.6	127.5	127.6
124.6	124.2	124.9	124.6	124.5	124.6	124.5	124.5	124.6
21.7	21.2	21.6	21.6	21.7	21.7	21.6	21.9	21.7
21.5	21.0	21.4	21.5	21.5	21.5	21.5	21.7	21.5

[a]Names correspond to those in the reference list. [b]"Disulfoxide."

Table S8. Comparison of ^{1}H NMR data (ppm (J Hz)) for *p*-TolSO_2STol-*p*[a] and Tol "disulfoxide"

Souto, Tol "disulfoxide"	Nair	Iwata	Shyam	This work
7.49 (d 8.4) 2H	7.56 (d 8.2) 2H	7.46 (d 8.0) 2H	7.46 (dd, 8.4,1.6) 2H	7.59 (d 8.3) 2H
7.29-7.16 (m) 4H	7.42 (d 8.1) 2H	7.24 (d 8.0) 2H	7.25-7.9 (m), 4H	7.46 (d 8.0) 2H
	7.29 (d 8.0) 2H	7.21 (d 8.0) 2H		7.33 (d 7.9) 2H
7.17 (d 8.0) 2H	7.18 (d 7.9) 2H	7.14 (d 8.0) 2H	7.14, (d 8.0) 2H	7.22 (d 7.8), 2H
2.45, s, 3H	2.42, s, 3H	2.42, s, 3H	2.42, s, 3H	2.44, s, 3H
2.41, s, 3H	2.38, s, 3H	2.38, s, 3H	2.37, s, 3H	2.41. s, 3H

[a]Names correspond to those in the reference list.

Table S9. Comparison of ^{13}C NMR data (ppm) for MesSO_2SMes and Mes "disulfoxide"

Souto, Mes "disulfoxide"	Zheng, 2016	Ji, 2021	This work
144.6	145.4	145.3	145.4
141.7	143.5	143.4	143.4
140.7	141.8	141.8	141.8
137.7	139.8	139.7	139.8
131.9	139.5	139.4	139.5
130.6	131.9	131.9	131.9
129.5	129.5	129.4	129.5
xx	123.4	123.3	123.4
23.5	22.7	22.6	22.7
21.5	21.5	21.4	21.5
21.4	21.3	21.2	21.2
19.1	21.1	21.0	21.1

[a]Names correspond to those in reference list.

Table S10. Comparison of ^{1}H NMR data (ppm) for MesSO_2SMes[a] and Mes "disulfoxide"

Souto, Mes "disulfoxide"	Zheng	Ji	This work
7.01	6.92	6.90	6.92
6.91	6.91	6.88	6.91
2.66	2.37	2.35	2.38
2.56	2.32	2.30	2.32
2.33	2.30	2.27	2.30
2.32	2.18	2.15	2.18

[a]Names correspond to those in reference list.

Table S11. Comparison of IR data (cm^{-1}) for *p*-$TolSO_2STol$[a,b] and Tol "disulfoxide"

Souto, Tol "disulfoxide"	Cai, 2010	Nair, 2003	Iwata, 2013	Shyam, 2016	This work
3012			3034		3035
2926		2915	2917		2915
			2862		2850
1596		1593	1590		1590
1491		1485	1488	1487	1488
1328	1333	1330	1323	1322	1322
1144	1143	1142	1138	1138	1135
		818	806		805

[a]Names correspond to those in reference list. [b]For IR spectra of $(TolS)_2$, TolS(O)STol, $TolSO_2STol$, $TolSO_2S(O)Tol$, $TolSO_2SO_2Tol$, and $(TolSO_2)_2$ O, see Bredereck, 1960.

Conclusion: The ^{1}H, ^{13}C and IR data for the Stockman group's tolyl and mesityl disulfide dioxides and data for authentic thiosulfonates from this work and from other laboratories is markedly similar, while there is good agreement for analogous data for the respective disulfides and thiosulfinates. The above data are inconsistent with 1,2-disulfoxide structures.

Table S5-S11 References

(1) Bredereck, H.; Wagner, A.; Beck, H.; Klein, R.-J. Die Struktur der Sulfinsäureanhydride. *Chem. Ber*., **1960**; *93*, 2736–2742; https://doi.org/10.1002/cber.19600931145.

(2) Cao, L.; Luo, S.-H.; Jiang, K.; Hao, Z.-F.; Wang, B.-W.; Pang, C.-M.; Wang, Z.-Y. Disproportionate Coupling Reaction of Sodium Sulfinates Mediated by $BF_3{\cdot}OEt_2$: An Approach to Symmetrical/Unsymmetrical Thiosulfonates. *Org. Lett.* **2018**, 20, 4754–4758; https://doi.org/10.1021/acs.orglett.8b01808.

(3) Ji, Y.-Z.; Zhang, C.; Wang, J.-H.; Li, H.-J.;Wu, Y.-C. Direct Conversion of Sulfinamides to Thiosulfonates Without the use of Additional Redox Agents Under Metal-Free Conditions. *Org. Biomol. Chem.* **2021**, *19*, 9291-9298; https://doi.org/10.1039/D1OB01714D.

(4) Zheng, Y.; Qing, F. L.; Huang, Y. G.; Xu, X. H. Tunable and Practical Synthesis of Thiosulfonates and Disulfides from Sulfonyl Chlorides in the Presence of Tetrabutylammonium Iodide. *Adv. Synth. Catal.* **2016**, *358*, 3477–3481; https://doi.org/10.1002/adsc.201600633.

(5) Shyam, P. K.; Kim, Y. K.; Lee, C.; Jang, H.-Y. Copper-Catalyzed Aerobic Formation of Unstable Sulfinyl Radicals for the Synthesis of Sulfinates and Thiosulfonates. *Adv. Synth. Catal.* **2016**, *358*, 56–61; https://doi.org/10.1002/adsc.201500785.

(6) Iwata, S.; Senoo, M.; Hata, T.; Urabe, H. Synthesis of *S*-Aryl Arenethiosulfonates from *N*,*N*-Di(arenesulfonyl)hydrazines: Reduction of Sulfonyl Chlorides with an Organic Reagent. *Heteroatom Chem.* **2013**, *24*, 336–344; https://doi.org/10.1002/hc.21088.

(7) Nair, V.; Augustine, A. Novel Synthesis of 2-Arylbenzothiazoles Mediated by Ceric Ammonium Nitrate(CAN): A Rebuttal. *Org. Lett.* **2003**, *5*, 543–544; https://doi.org/10.1021/ol027452w.

(8) Cai, M.-T.; Lv. G.-S.; Chen, J.-X.; Gao, W.-X.; Ding, J.-C.; Wu, H.-Y. CAN/I_2-catalyzed Chemoselective Synthesis of Thiosulfonates by Oxidation of Disulfides or Thiols. *Chem. Lett.* **2010**, *39*, 368–369; https://doi.org/10.1246/cl.2010.368.

Historical Summary of 1,2-Disulfoxide, RS(O)S(O)R, and Relationship to Thiosulfonates, RSO_2SR

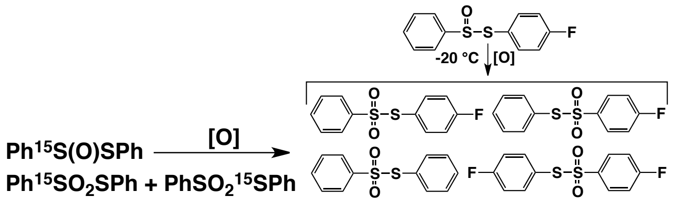

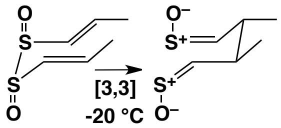

BLYPT calculations: weak 20 kcal/mol S–S bond in MeS(O)S(O)Me

1865-8

Otto/Märcker *Ann*. **1865**, *136*, 75; **1868**, *145*, 317. Early reports of synthesis of $Ar_2S_2O_2$; a diaryl "disulfoxide" ArS(O)S(O)Ar?

Otto, *Chem. Ber*. **1876**, *9*, 1636 $Ar_2S_2O_2 + ArS^- \rightarrow ArSO_2^- + ArSSAr$

Smiles, *JCS* **1924**, *125*, 176; chemistry favors $ArSO_2SAr$; Gilman, *JACS*, **1925**, *47*, 851 chemistry favors $ArSO_2SAr$; Lavine, *J. Biol. Chem.* **1936**, *113*, 571, 583, cystine α-disulfoxide?

Cymerman, JCS, **1951**, 1332; IR of ArS(O)Ar at 1040 cm^{-1}. $Ar_2S_2O_2$ shows SO_2 bands at 1150 and 1340 cm^{-1} favoring $ArSO_2SAr$. Similar results: S.S. Block *Appl. Spec.* **1966**, *20*, 73; IR and Raman confirm RSO_2SR for $R_2S_2O_2$. Our IR spectrum of $ArSO_2SAr$:

Barnard, *JCS*, **1957**, 4673-5 RS(O)Cl + Zn → RSO_2SR (not RS(O)S(O)R); ArS(O)SAr → $ArSO_2SAr$ + ArSSAr; *JCS*, **1957**, 4675-6 *Chem. Ind.* **1960**, 1332 $Ar^{15}S(O)SAr + H_2O_2 \rightarrow Ar^{15}SO_2SAr + ArSO_2{}^{15}SAr$

Fava, *Chem. Commun.* **1965**, 417; ArS(O)SAr → $ArSO_2SAr$ + ArSSAr Axelson, *Spectrochim Acta* **1967** *23A* ESCA = cyst-SO_2S- not α-disulfoxide

Kice, *JACS*, **1976**, *98*, 7711; low temp. NMR of oxidation of PhS(O)SAr shows PhS(O)S(O)Ar with $t_{1/2}$ at -20 °C ≤ 60 sec; weak S–S bond; "extraordinarily unstable" compound. Oae, *BCSJ* **1982**, 55, 2484.

Freeman, *JACS*, **1981**, *103*; 6232; **1982**, *104*, 1161; 5766; **1983**, *105*, 4039; *Chem. Rev.* **1984**, *84*, 117; low temp. NMR study of oxid. of tBuS(O)SBut shows. tBuS(O)S(O)But & [tBuS(O)]$_2$O; no isolations of disulfoxides at rt; calc. for HS(O)S(O)H

E. Block, *JACS*, **1990**, *112*, 4584; **1996**, 118, 2799; *vic*-disulfoxides in onion extracts generated synthetically rearrange at -20 °C. *JACS*, **1980**, *102*, 2490: a sulfenyl sulfinate gives a thiosulfonate

Harpp, *JACS*, **1993**, *115*, 3066. First isolation of cyclic α-disulfoxide, stable at 30 °C; also *JOC*, **1995**, *60*, 1044. Also see Grainger *Angew. Chem. Int. Ed.* **2009** *48*, 4832; Ishii, *JACS*, **1999** *121*, 7959; *Org. Lett*., **2006**, 91, **2004**, 2623.

Jenks, *J. Phys. Chem. A* **2003**, *107*, 3414, Calc. for MeS(O)S(O)Me show very weak S–S bond, ΔH = 20 kcal mol^{-1}

p$TolSO_2STol$*p* structure confirmed by X-ray crystallography: Caputo, **1984**; Ferguson, **2000**; Liang, **2012**; Cao, **2018**; Chen, **2024**

present

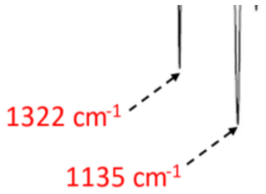

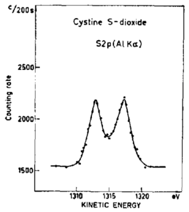

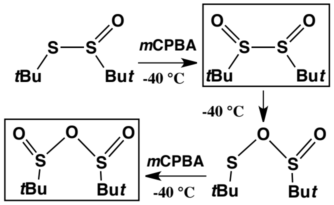

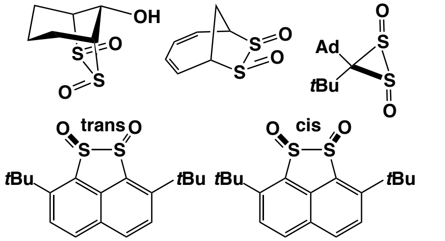

References to historical summary

- Axelson, G.; Hamrin, K. Fahlman, A. Nordling C. Lindberg, B. J. Electron Spectroscopic Evidence of the Thiolsulphonate Structure of Cystine *S*-Dioxide. *Spectrochim. Acta Part A: Mol. Spectros.* **1967**, *23*, 2015–2020; https://doi.org/10.1016/0584-8539(67)80089-8.
- Barnard, D. The Reaction of Sulphinyl Chlorides with Zinc. *J. Chem. Soc.* **1957a**, 4673–4675; https://doi.org/10.1039/JR9570004673.
- Barnard, D. The Spontaneous Decomposition of Aryl Thiolsulphinates. *J. Chem. Soc.* **1957b**, 4675–4676; https://doi.org/10.1039/JR9570004675.
- Barnard, D.; Percy, E. J. Oxidation of Thiosulphinates to Thiolsulphonates. *Chem. Ind.* **1960**, 1332–1333.
- Block, E.; Bayer, T. "(*Z,Z*)-*d,l*-2,3-Dimethyl-1,4-butanedithial 1,4-Dioxide: A Novel Biologically Active Organosulfur Compound from Onion. Formation of *vic*-Disulfoxides in Onion Extracts", *J. Am. Chem. Soc.* **1990**, *112*, 4584-4585; https://doi.org/10.1021/ja00167a089.
- Block, E. Bayer, T. Naganathan, S. Zhao, S.-H. *Allium* Chemistry: Synthesis and Sigmatropic Rearrangements of Alk(en)yl 1-Propenyl Disulfide *S*-Oxides from Cut Onion and Garlic, *J. Am. Chem. Soc.*, **1996**, *118*, 2799-2810; https://doi.org/10.1021/ja953444h
- Block, S. S.; Weidner, J. P. Vibrational Behavior and Structure of Disulfide Dioxides (Thiosulfonates). *Appl. Spec.* **1966**, *20*, 73–79; https://doi.org/10.1366/000370266774386627.
- Cao, L.; Luo, S.-H.; Jiang, K.; Hao, Z.-F.; Wang, B.-W.; Pang, C.-M.; Wang, Z.-Y. Disproportionate Coupling Reaction of Sodium Sulfinates Mediated by $BF_3 \cdot OEt_2$: An Approach to Symmetrical/Unsymmetrical Thiosulfonates. *Org. Lett.* **2018**, *20*, 4754–4758; https://doi.org/10.1021/acs.orglett.8b01808. X-ray of $TolSO_2STol$
- Caputo, R.; Palumbo, G.; Nardelli, M.; Pelizzi, G. Structure and Conformation of Symmetric Aryl Thiosulphonic Esters. *Gazz. Chim. Ital.* **1984**, *114*, 421–430. X-ray of $TolSO_2STol$
- Chau, M. M.; Kice, J. L. A Search for an α-Disulfoxide as an Intermediate in the Oxidation of an Aryl Thiosulfinate. *J. Am. Chem. Soc.* **1976**, *98*, 7711–7716; https://doi.org/10.1021/ja00440a043.
- Chen, Q.; Chen, Z.-H.; Liang, Y.-T.; Zeng, Y.; Yu, S.-W.; Yang, K.; Wang. Z.-Y. I_2-promoted Disproportionate Coupling Reaction of Aryl Sulfonyl Hydrazides: A Simple and Green Access to Thiosulfonates. *Synthesis*, **2024**, *56*, 1415–1421; https://doi.org/10.1055/a-2248-5438. X-ray of $TolSO_2STol$
- Cymerman, J.; Willis, J. B. The Infra-red Spectra and Chemical Structure of Some Aromatic Disulphides, Disulphones, and Thiolsulphonates. *J. Chem. Soc.* **1951**, 1332–1337; https://doi.org/10.1039/JR9510001332.
- Derbesy, G.; Harpp, D. N. Detection and Decomposition of Di-*tert*-butyl Disulfide–Polyoxide Derivatives. *J. Org. Chem.* **1995**, *60*, 1044–1052; https://doi.org/10.1021/jo00109a042.
- Ferguson, G.; Glidewell, C.; Low, J. N.; Wardell, J. L. A Second Monoclinic Polymorph of *ƒ*-(4-Tolyl) 4-Toluenethiosulfonate at 150 and 293 K. *Acta Cryst.* **2000**, *C56*, 692–694; https://doi.org/10.1107/S0108270100003280. X-ray of $TolSO_2STol$
- Folkins, P. L.; Harpp, D. N. α-Disulfoxides and Other Intermediates in the Oxidation of Disulfides. *J. Am. Chem. Soc.* **1993**, *115*, 3066–3070; https://doi.org/10.1021/ja00061a004.

- Freeman, F.; Angeletakis, C. N. α-Disulfoxide and Sulfinic Anhydride in the Peroxy acid Oxidation of 2-Methyl-2-propyl 2-Methyl-2-propanethiosulfinate. *J. Am. Chem. Soc.* **1981**, *103*, 6232–6235; https://doi.org/10.1021/ja00410a051.
- Gilman, H.; Smith, L. E.; Parker, H. H. The Constitution of Disulfoxides. *J. Am. Chem. Soc.* **1925**, 47, 851–860; https://doi.org/10.1021/ja01680a037.
- Gregory, D. D.; Jenks, W. S. Computational Investigation of Vicinal Disulfoxides and Other Sulfinyl Radical Dimers. *J. Phys. Chem. A* **2003**, *107*, 3414-3423; https://doi.org/10.1021/jp026888q.
- Grainger, R. S.; Patel, B.; Kariuki, B. M. 2,7-Di-*tert*-butylnaphtho[1,8-*cd*][1,2]dithiole 1,2-dioxides: Thermally Stable, Photochemically Active *vic*-Disulfoxides. *Angew. Chem. Int. Ed.* **2009**, *48*, 4832–4835; https://doi.org/10.1002/anie.200901788.
- Ishii, A.; Kashiura, S.; Oshida, H.; Nakayama, J. First Isolation of Eclipsed *vic*-Disulfoxide: 7,8-Dithiabicyclo[4.2.1]nona-2,4-diene 7-*exo*,8-*exo*-Dioxide. *Org. Lett.* **2004**, *6*, 2623–2626; https://doi.org/10.1021/ol049031f.
- Ishii, A.; Nakabayashi, M.; Nakayama, J. 1-Adamantyl-*tert*-butyltetrathiolane 2,3-Dioxide: First Isolable *vic*-Disulfoxide and Efficient Precursor of S_2O. *J. Am. Chem. Soc.* **1999**, *121*, 7959–7960; https://doi.org/10.1021/ja9915955.
- Ishii, A.; Ohishi, M.; Matsumoto, K.; Takayanagi, T. Synthesis and Properties of a Dithiirane *trans*-1,2-Dioxide, a Three-Membered *vic*-Disulfoxide. *Org. Lett.* **2006**, *8*, 91–94; https://doi.org/10.1021/ol052570f.
- Koch, P.; Ciuffarin, E.; Fava, A. Thermal Disproportionation of Aryl Arenethiosulfinates. Kinetics and Mechanism. *J. Am. Chem. Soc.* **1971**, *92*, 5971–5977; https://doi.org/10.1021/ja00723a026.
- Lavine, T. F. The Oxidation of Cystine in Non-aqueous Media. VI. A Study of the Reactions of the Disulfoxide of Cystine, Especially of its Dismutative Decompositions. *J. Biol. Chem.* **1936**, *113*, 583–597; https://doi.org/10.1016/S0021-9258(18)74878-3; Toennies, G.; Lavine, T. F. The Oxidation of Cystine in Non-aqueous Media. V. Isolation of a Disulfoxide of Cystine. *J. Biol. Chem.* **1936**, *113*, 571–582; https://doi.org/10.1016/S0021-9258(18)74877-1.
- Liang, G.; Liu, M.; Chen, J.; Ding, J.; Gao, W.; Wu, H. NBS-Promoted Sulfenylation of Sulfinates with Disulfides Leading to Unsymmetrical or Symmetrical Thiosulfonates. *Chin. J. Chem.* **2012**, *30*, 1611–1616; https://doi.org/10.1002/cjoc.201200028. X-ray of $TolSO_2STol$
- Märcker, C. Ueber einige Schwefelhaltige Derivate des Toluols. *Justus Liebigs Annalen der Chemie*, **1865**, *136*(1), 75-95; https://doi.org/10.1002/jlac.18651360108.
- Oae, S.; Kim. Y. H.; Takata, T.; Fukushima, D. The Oxidation of Unsymmetrical Thiosulfinate: Evidence for α–Disulfoxide as an Intermediate. *Tetrahedron Lett.* **1977**, 1195–1198; https://doi.org/10.1016/S0040-4039(01)92868-0.
- Oae, S.; Takata, T.; Kim, Y. H. Oxidation of Unsymmetrical Disulfide and Thiosulfinic *S*-Esters with Peroxy Acids. Search for Formation of α-Disulfoxide as An Intermediate in the Electrophilic Oxidation of Thiosulfinic *S*-Ester. *Bull. Chem. Soc. Jpn.* **1982**, *55*, 2484–2494; https://doi.org/10.1246/bcsj.55.2484.
- Oae, S.; Yoshikawa, Y.; Tagaki, W. Alkaline Hydrolysis of Aryl Benzenethiosulfinates. *Bull. Chem. Soc. Jpn.* **1969**, *42*, 2899–2903; https://doi.org/10.1246/bcsj.42.2899.

- Otto, R. Ueber einige Derivate des Benzols und Toluols. *Justus Liebigs Annalen der Chemie*, **1868**, *145*(3), 317–329; https://doi.org/10.1002/jlac.18681450308.
- Schiller, R.; Otto, R. Versuche zur Darstellung von thioäthern der Benzolsulfonsäure_und Paratoluolsulfonsäure. *Chem. Ber.* **1876**, 1636–1637; https://doi.org/10.1002/cber.187600902180.
- Smiles, S.; Gibson, D. T. The Constitution of Disulfoxides. Part I. *J. Chem. Soc.* **1924**, *125*, 176–183; https://doi.org/10.1039/CT9242500176.
